# Unraveling Fungal Radiation Resistance Regulatory Networks through the Genome-Wide Transcriptome and Genetic Analyses of *Cryptococcus neoformans*

**DOI:** 10.1128/mBio.01483-16

**Published:** 2016-11-29

**Authors:** Kwang-Woo Jung, Dong-Hoon Yang, Min-Kyu Kim, Ho Seong Seo, Sangyong Lim, Yong-Sun Bahn

**Affiliations:** a Research Division for Biotechnology, Korea Atomic Energy Research Institute, Jeongeup, Republic of Korea; b Department of Biotechnology, College of Life Science and Biotechnology, Yonsei University, Seoul, Republic of Korea

## Abstract

The basidiomycetous fungus *Cryptococcus neoformans* has been known to be highly radiation resistant and has been found in fatal radioactive environments such as the damaged nuclear reactor at Chernobyl. To elucidate the mechanisms underlying the radiation resistance phenotype of *C. neoformans*, we identified genes affected by gamma radiation through genome-wide transcriptome analysis and characterized their functions. We found that genes involved in DNA damage repair systems were upregulated in response to gamma radiation. Particularly, deletion of recombinase *RAD51* and two DNA-dependent ATPase genes, *RAD54* and *RDH54*, increased cellular susceptibility to both gamma radiation and DNA-damaging agents. A variety of oxidative stress response genes were also upregulated. Among them, sulfiredoxin contributed to gamma radiation resistance in a peroxiredoxin/thioredoxin-independent manner. Furthermore, we found that genes involved in molecular chaperone expression, ubiquitination systems, and autophagy were induced, whereas genes involved in the biosynthesis of proteins and fatty acids/sterols were downregulated. Most importantly, we discovered a number of novel *C. neoformans* genes, the expression of which was modulated by gamma radiation exposure, and their deletion rendered cells susceptible to gamma radiation exposure, as well as DNA damage insults. Among these genes, we found that a unique transcription factor containing the basic leucine zipper domain, named Bdr1, served as a regulator of the gamma radiation resistance of *C. neoformans* by controlling expression of DNA repair genes, and its expression was regulated by the evolutionarily conserved DNA damage response protein kinase Rad53. Taken together, the current transcriptome and functional analyses contribute to the understanding of the unique molecular mechanism of the radiation-resistant fungus *C. neoformans*.

## IMPORTANCE

Although there are no natural environments under intense radiation, some living organisms have been found to show high radiation resistance. Organisms harboring the ability of radiation resistance have unique regulatory networks to overcome this stress. *Cryptococcus neoformans* is one of the radiation-resistant fungi and is found in highly radioactive environments. However, it remains elusive how radiation-resistant eukaryotic microorganisms work differentially from radiation-sensitive ones. Here, we performed transcriptome analysis of *C. neoformans* to explore gene expression profiles after gamma radiation exposure and functionally characterized some of identified radiation resistance genes. Notably, we identified a novel regulator of radiation resistance, named Bdr1 (a bZIP TF for DNA damage response 1), which is a transcription factor (TF) that is not closely homologous to any known TF and is transcriptionally controlled by the Rad53 kinase. Therefore, our work could shed light on understanding not only the radiation response but also the radiation resistance mechanism of *C. neoformans*.

## INTRODUCTION

Exposure to ionizing radiation (IR) from natural sources or caused by human activities damages the cellular components of all living organisms, including nucleic acids, proteins, and lipids, through both direct energy deposition of IR and interaction with reactive oxygen species (ROS), such as hydroxyl radicals (OH⋅), superoxide anions, and hydrogen peroxide, generated by radiolysis of water. For instance, various types of DNA lesions, including base modification, abasic sites, and strand breaks, are caused by the interaction of OH⋅ with DNA and direct ionization of the DNA molecules, normally resulting in detrimental effects on cell survival (1). To counteract these fatal effects, cells activate arrays of DNA repair machineries and antioxidative defense systems, and if the IR-induced damage exceeds the capacity of cells to repair it, the cells die. Exposure to 200 Gy (i.e., “grays,” the SI unit of absorbed radiation dose) is lethal to most bacteria (2). Interestingly, however, radiation-resistant organisms, which are capable of withstanding high doses of radiation (5 to 10 kGy) without loss of viability, have been found in three domains of life (3). The IR defense systems of these organisms provide novel molecular insights into the mechanism of ROS detoxification and DNA repair processes.

In eubacteria, *Deinococcus radiodurans*, which is ubiquitously found in soil, is the best known radiation-resistant bacterium that is able to survive high doses of gamma radiation, 20 times greater than those of the bacterium *Escherichia coli*: the radiation dose yielding 10% survival (D_10_) of *D. radiodurans* is 12 kGy, whereas that of *E. coli* is 0.2 to 0.7 kGy (4). *D. radiodurans* has various DNA repair systems, including extended synthesis-dependent strand annealing and the RecF pathway of homologous recombination (HR), which can efficiently repair DNA double-strand breaks (DSBs), considered to be the most lethal form of damage. This organism removes ROS through enzymatic systems, such as superoxide dismutase, catalase, and peroxidase, and nonenzymatic systems, such as pyrroloquinoline-quinone, deinoxanthin, and bacillithiol (5, 6). In particular, the unusual Mn^2+^ accumulation in *D. radiodurans*, which results in a high intracellular Mn/Fe ratio, has been correlated with IR resistance through the formation of low-molecular-weight ROS-scavenging Mn^2+^-metabolite complexes (2, 4). It is interesting to note that a high Mn/Fe ratio is observed in other radiation-resistant bacteria, such as *Rubrobacter radiotolerans* (D_10_, 12 kGy) and *Kineococcus radiotolerans* (D_10_, 3 kGy) (7, 8). The halophilic archaeon *Halobacterium salinarum* NRC1 showed remarkable IR resistance (D_10_, 5 kGy) (9). When *H. salinarum* was exposed to IR, DNA repair was primarily mediated by HR and glycosylase activity, in which single-stranded DNA-binding proteins (called replication proteins A [RPA]) played key roles (10, 11). Not only a high Mn/Fe ratio but also a high halide concentration in the cytoplasm of *H. salinarum* provided a measure of protection for its macromolecules against the oxidative effects of IR (12, 13). In contrast, the hyperthermophilic archaea *Thermococcus gammatolerans* (D_10_, 6 kGy) and *Pyrococcus furiosus* (D_10_, 3 kGy) do not contain significant amounts of intracellular Mn (8). Instead, they are equipped with numerous detoxification systems to cope with the ROS produced by IR (14, 15).

In eukaryotes, the DNA repair systems of the phytopathogenic fungus *Ustilago maydis* (D_10_, 3.6 kGy) have been studied to explain IR resistance (16). HR machinery is known to contribute to the gamma radiation resistance of *U. maydis* (17, 18). *BRH2*, a functional homolog of the *BRCA2* (breast cancer 2) gene in humans, is a key component of the HR system of *U. maydis* (18). Dss1, which is a small acidic protein that interacts with Brh2, is necessary for Brh2 activity. Deletion of Brh2 or Dss1 results in radiation sensitivity and recombination deficiency of *U. maydis* (19–21). Although these two proteins contribute to the gamma radiation resistance of *U. maydis*, the presence of BRCA2 or Dss1 orthologs in radiation-sensitive vertebrates and eukaryotes (22–24) indicates that other factors involved in radioresistance remain uncharacterized.

The basidiomycetous fungal pathogen *Cryptococcus neoformans*, which causes fatal meningoencephalitis in humans (25), was found to be a dominant species in highly radioactive environments, such as the cooling pools of nuclear reactors, the stratosphere, and the damaged nuclear reactor at Chernobyl (26). The radiation resistance mechanism of *C. neoformans* has been studied from the aspect of melanin production. Dadacohva et al. demonstrated that melanized cryptococcal cells exhibited increased growth using the enhanced electron transfer properties of melanin after exposure to IR, compared to nonmelanized cells (27). Furthermore, melanin quenches IR-induced ROS, thereby preventing subsequent DNA damage (28). However, there has been no systematic and comprehensive approach to elucidating the radiation resistance mechanism of *C. neoformans*.

In this study, we performed a DNA microarray-based transcriptome analysis of the *C. neoformans* var. *grubii* H99 strain, which is a serotype A genome sequencing platform strain, to explore gene expression profiles during the postirradiation period, and we identified genes underlying the IR resistance phenotype of *C. neoformans* by reverse-genetics approaches. Notably, we functionally characterized a unique radiation response bZIP transcription factor (TF), Bdr1 (a bZIP TF for DNA damage response 1), which regulates expression levels of genes involved in DNA repair systems, and we found that its transcription level was controlled by the Rad53 protein kinase. This study could help us to understand the genome-wide radiation resistance networks and mechanism in the basidiomycetous fungi as well as *C. neoformans*.

## RESULTS

### Intrinsic cellular factors contribute to radiation resistance of *Cryptococcus neoformans* in addition to melanin pigment.

**FIG 1:**
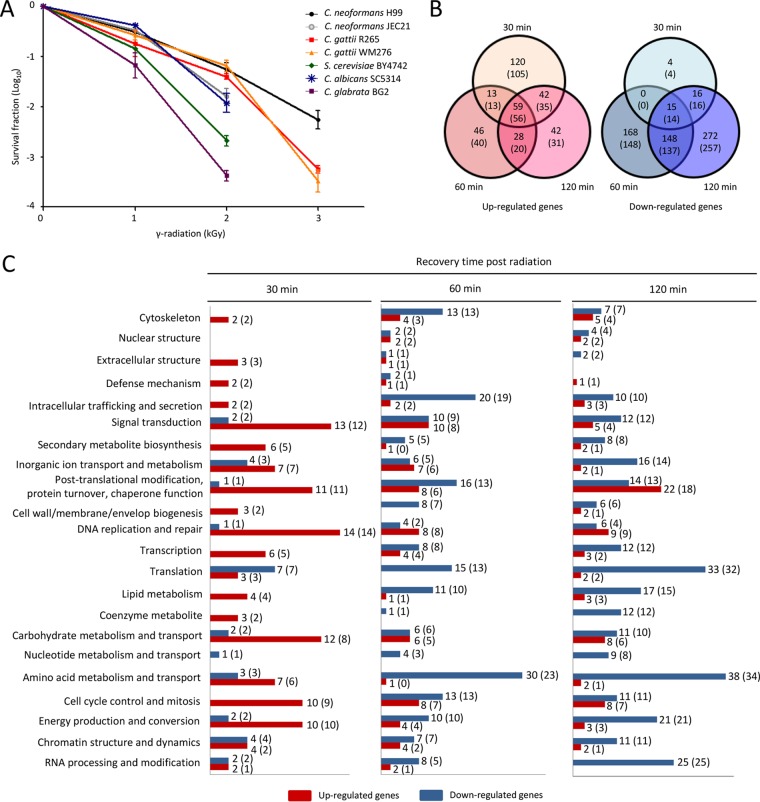
Comparative transcriptome analysis of the *C. neoformans* H99 strain in response to gamma radiation. (A) Each fungal species was grown in liquid YPD medium at 30°C overnight. Cells were exposed to the indicated dose of gamma radiation for 1 h. Next, cells were serially diluted (1 to 10^4^ dilution), spread on solid YPD medium, and further incubated at 30°C for 3 days. The survival fraction was determined by comparison with nonirradiated cells of each corresponding strain. (B) Venn diagrams exhibiting the number of upregulated (2-fold) and downregulated (2-fold) genes at 30, 60, and 120 min post-gamma radiation exposure. The number of genes was determined for genes with expression levels changed more or less by 2-fold (*P* < 0.05; ANOVA). (C) Functional categories of radiation-responsive genes in *C. neoformans*. Among the radiation-responsive genes, genes exhibiting more than 2-fold changes were categorized based on the KOG functional description (eukaryotic orthologous group; http://www.ncbi.nlm.nih.gov/COG). The number of genes in parentheses was determined for genes with expression levels changed more or less by 2-fold (*P* < 0.01; ANOVA). The red and blue bars represent the number of genes upregulated and downregulated, respectively, by radiation exposure.

Although *C. neoformans* has been known to be a radiation-tolerant fungus, the ability of the pathogenic *Cryptococcus* species (PCS) complex to survive radiation has not yet been analyzed in detail. Therefore, we compared the radiation resistance levels of the PCS complex (the *C. neoformans* var. *grubii* H99 strain, *C. neoformans* var. *neoformans* JEC21 strain, *Cryptococcus gattii* R265 strain, and *C. gattii* WM276 strain) with those of ascomycete nonpathogenic model yeast *Saccharomyces cerevisiae* (BY4742) and the pathogenic yeasts *Candida albicans* (SC5314) and *Candida glabrata* (BG2). Compared to the *S. cerevisiae* and pathogenic *Candida* species, the PCS complex, except for the var. *neoformans* strain, generally exhibited increased resistance to gamma radiation (Fig. 1A). Among PCS complexes, the *C. neoformans* var. *grubii* (H99) strain was most tolerant to gamma radiation, and *C. gattii* strains were more tolerant to gamma radiation than the *C. neoformans* var. *neoformans* strain (JEC21).

Melanized *C. neoformans* and *Histoplasma capsulatum* are more resistant to gamma radiation than their nonmelanized cells (28, 29). Therefore, we wondered whether the expression levels of laccase genes (*LAC1* and *LAC2*) involved in producing melanin were increased after radiation exposure. We demonstrated that the expression patterns of melanin-producing genes *LAC1* and *LAC2* were gradually decreased after high (3 kGy) or low (1 kGy) doses of gamma radiation exposure (see Fig. S1 in the supplemental material), suggesting that gamma radiation itself did not trigger melanin formation. This phenomenon was in stark contrast to the finding that *LAC1* and *LAC2* were greatly increased either during oxidative stress responses (30) or by carbon starvation (31).

### *C. neoformans* significantly remodels transcriptome profiles in response to gamma radiation.

The fact that transcript abundance of *LAC1* and *LAC2* was decreased during postradiation recovery led us to examine alternative mechanisms to endowing *C. neoformans* var. *grubii* (hereafter described as *C. neoformans*) with radiation resistance. To this end, we monitored genome-wide transcriptional patterns in response to gamma radiation by performing a DNA microarray-based transcriptome analysis of the H99 strain. To elucidate changes in transcriptome profiles during postradiation recovery, cells with or without exposure to gamma radiation (3 kGy for 1 h) were allowed to recover for 30, 60, or 120 min under nonradiation conditions and were used for total RNA isolation. To obtain high reliability in the array data, three independent DNA microarrays with three independent biological replicates were analyzed.

The transcriptome analysis revealed that a total of 2,587 *C. neoformans* genes displayed different expression patterns in response to gamma radiation (*P* < 0.05; 2,016 genes at *P* < 0.01 by analysis of variance [ANOVA]), suggesting that a considerable proportion of *C. neoformans* genes (37% of a total of 6,962 genes) were transcriptionally regulated during recovery from gamma radiation exposure. Totals of 234, 146, and 171 genes (209, 129, and 142 genes at *P* < 0.01) exhibited more than 2-fold induction at different recovery time points (30, 60, and 120 min, respectively). Among these genes, 59 genes (56 genes at *P* < 0.01) were upregulated by more than 2-fold at all of the time points. Similarly, totals of 35, 331, and 451 genes (34, 299, and 424 genes at *P* < 0.01) were downregulated by more than 2-fold at different recovery time points (30, 60, and 120 min, respectively). The expression level of 15 genes (14 genes at *P* < 0.01) decreased more than 2-fold at all time points (Fig. 1B). The Pearson correlation coefficient (PCC) between the DNA microarray-based transcriptome analysis and quantitative reverse transcriptase PCR (qRT-PCR) data (PCC = 0.8321) indicated that microarray data and qRT-PCR data were highly correlated, further supporting the quality of our analysis.

The *C. neoformans* gamma radiation-responsive genes were assorted using the KOG (eukaryotic orthologous group) classification. At an early recovery time (30 min), genes involved in DNA replication and repair, signal transduction, and posttranslational modification and chaperone functions were induced, suggesting that cells immediately activated defense systems to counteract the effects of gamma radiation (Fig. 1C). In contrast, a number of genes involved in amino acid metabolism and transport, RNA processing and modification, and translation were significantly downregulated at a later time points (60 and 120 min), indicating that cells attempted to curtail basic cellular function to avoid the toxic effects resulting from the production of abnormal proteins, fatty acids/sterols, and other cellular molecules (Fig. 1C). Taken together, *C. neoformans* cells extensively remodeled transcriptome profiles in response to gamma radiation to counteract any direct cellular damage and to avoid indirect toxic effects caused by radiation.

### Recombinase, Rad51, and two DNA-dependent ATPases, Rad54 and Rdh54, play critical roles in the survival of *C. neoformans* upon exposure to gamma radiation.

To counteract the adverse effects of radiation, cells induced the expression of genes involved in DNA repair systems (32). In agreement with previous transcriptome analyses of data in ascomycete model yeasts (33, 34), our transcriptome data also revealed that genes for DNA repair systems were highly induced upon radiation exposure, indicating that increasing genome integrity is a common cellular response to gamma radiation among fungi (see Table S3 in the supplemental material).

**FIG 2:**
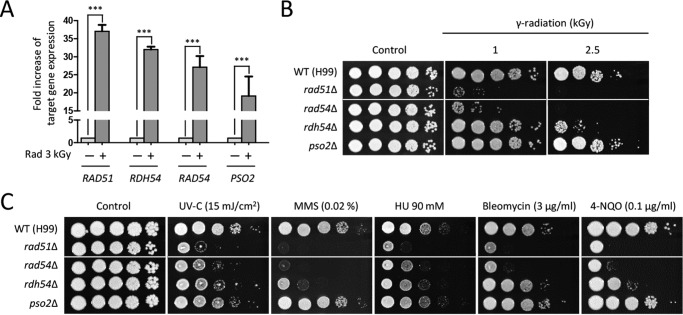
DNA repair response is critical for gamma radiation resistance. (A) Fold increase in expression of DNA repair-related genes exposed to gamma radiation. The fold increase of target gene expression was quantitatively measured by qRT analysis using the gene-specific primers listed in Table S2. The cDNA was synthesized with total RNAs extracted from cells recovered at 30 min after exposure to gamma radiation or not exposed to gamma radiation. Duplicate technical experiments with two or more biological samples were performed. Representative images from independent experiments for each DNA damage-responsive gene are shown. Error bars indicate standard deviations. Asterisks indicate the statistical significance of differences in expression levels of each gene (***, *P* < 0.001). (B) Spotting assay for gamma radiation resistance. Cells cultured overnight in liquid YPD medium were serially spotted onto the solid YPD medium and then exposed to the indicated dose of gamma radiation. Exposed cells were further incubated at 30°C and photographed for 1 to 3 days. (C) Rdh54, Rad54, and Rig1 are required for DNA damage response in *C. neoformans*. The wild-type (WT [H99]) or *rad51*Δ (KW362), *rdh54*Δ (KW78), *rad54*Δ (KW26), and *pso2*Δ (KW22) mutant *C. neoformans* strains were grown overnight at 30°C in liquid YPD medium, and the 10-fold serially diluted cells were spotted onto YPD agar containing the indicated concentrations of genotoxic DNA damage insults. Cells were incubated at 30°C and photographed for 1 to 3 days. The two images split by a horizontal white line in each spot assay were obtained from the same plate (B and C).

To verify the transcriptome data, we measured the expression levels of some of the DNA-damage-responsive genes using quantitative reverse transcriptase PCR (qRT-PCR) analysis. We demonstrated that the expression levels of *RAD51* (a recombinase and a homolog of bacterial RecA protein), *RAD54* (a DNA-dependent ATPase), *RDH54* (a DNA-dependent ATPase), and *PSO2* (a nuclease required for DNA single- and double-strand break repair) genes were significantly increased during gamma radiation (Fig. 2A). To verify accurate phenotypes caused by target gene deletion and exclude unexpected mutational effects, we constructed two independent deletion strains for each gene and confirmed identical phenotypes of these two mutants in response to gamma radiation resistance as well as DNA damage insults (data not shown). The *rad51*Δ, *rdh54*Δ, and *rad54*Δ mutants exhibited severe growth defects in response to gamma radiation, whereas the *pso2*Δ mutant showed wild-type (WT) levels of gamma radiation resistance (Fig. 2B).

Given that *RAD51*, *RDH54*, *RAD54*, and *PSO2* are involved in the DNA repair system in *S. cerevisiae* (35–38), we determined whether these genes were required for counteracting DNA damage stresses other than gamma radiation. The *rad51*Δ and *rad54*Δ mutants exhibited highly increased susceptibility to other DNA damage stress inducers, including UV irradiation, methyl methanesulfonate (MMS), hydroxyurea (HU), bleomycin, and 4-nitroquinoline *n*-oxide (4-NQO) (Fig. 2C). The *rdh54*Δ mutant exhibited susceptibility to UV irradiation, MMS, HU, and 4-NQO. The *rad54*Δ mutant displayed greater growth defects in response to DNA-damaging stresses than the *rdh54*Δ mutant, suggesting that Rad54 plays a more significant role in DNA damage repair than Rdh54. However, the *pso2*Δ mutant showed wild-type levels of resistance to genotoxic stresses (Fig. 2C).

### Sulfiredoxin is required for the survival of *Cryptococcus neoformans* under gamma radiation exposure in a peroxiredoxin-independent manner.

**FIG 3:**
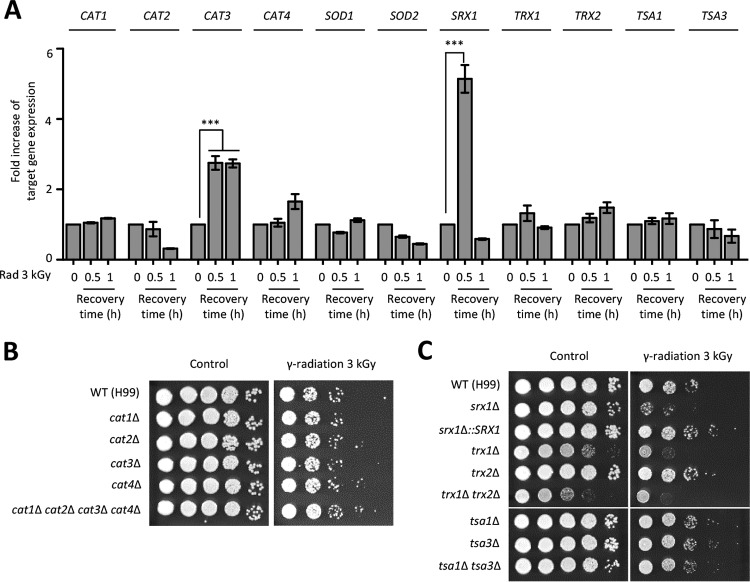
The oxidative response system is required for gamma radiation resistance. (A) Fold increase in expression of genes involved in oxidative stress response after radiation exposure. The expression patterns of target genes were quantitatively determined using qRT analysis with the gene-specific primers listed in Table S2. To determine the expression levels of target genes, the cDNA was synthesized with total RNAs extracted from cells recovered 30 and 60 min after exposure to gamma radiation (3 kGy) or not exposed to gamma radiation. Duplicate technical experiments with three biological samples were performed. Representative images from independent experiments for each target gene are shown. Error bars indicate standard deviations. Asterisks indicate the statistical significance of differences in expression levels of each gene (***, *P* < 0.001). (B and C) Each *Cryptococcus* strain was cultured in the liquid YPD medium at 30°C overnight. Serially diluted (1 to 10^4^) cells were spotted onto the YPD medium and then exposed to the indicated dose of gamma radiation. The cells were further incubated at 30°C for 2 days and photographed daily. The two images split by a horizontal white line in each spot assay were obtained from the same plate (C).

In addition to genome instability, radiation indirectly causes acute and transient intracellular oxidative stress through free radicals generated from water (39). Supporting this notion, our transcriptome analysis showed that a number of oxidative stress defense genes were highly upregulated in response to gamma radiation. To confirm this finding further, we examined the expression levels of these oxidative responsive genes, such as the genes coding for superoxide dismutases (*SOD1* and *SOD2* [converting superoxide anion to hydrogen peroxide]), catalases (*CAT1*, *CAT2*, *CAT3*, and *CAT4* [converting hydrogen peroxide to water]), peroxiredoxins (*TSA1* and *TSA3* [reducing hydrogen peroxide using the thioredoxin system]), thioredoxins (*TRX1* and *TRX2* [acting as electron donors to peroxidase]), and sulfiredoxin (*SRX1* [recycling the sulfinic acid form of peroxiredoxin to its sulfenic acid form in an ATP-dependent reaction]). Among these genes, expression of *SRX1* was most dramatically increased at 30 min post-radiation exposure and then decreased to a basal level at 60 min. Among the four catalases, expression of *CAT3* was increased during recovery from radiation exposure. However, the expression levels of superoxide dismutases, peroxiredoxin, and thioredoxin systems were not significantly changed during recovery after radiation treatment (Fig. 3A).

Next, we constructed strains with each catalase gene deleted and monitored their radiation resistance relative to those of strains lacking sulfiredoxin, peroxiredoxin, and thioredoxin, which have been reported previously (40). Although the expression level of *CAT3* was induced, the *cat3*Δ mutant showed wild-type levels of radiation resistance (Fig. 3B). Moreover, the *cat1*Δ *cat2*Δ *cat3*Δ *cat4*Δ quadruple mutant, which was previously reported (41), was as resistant to gamma radiation as the wild-type H99 strain and each catalase single mutant (Fig. 3B). In agreement with the strong induction of *SRX1* expression, however, deletion of *SRX1* caused severe growth defects upon radiation exposure, while its complemented strain (*srx1*Δ::*SRX1*) exhibited wild-type levels of radiation resistance (Fig. 3C). However, deletions of *TRX1*, *TRX2*, *TSA1*, and *TSA3* did not alter radiation resistance significantly (Fig. 3C). These results suggested that Srx1 was required for the gamma radiation resistance that occurred in a peroxiredoxin-independent manner.

### Protein quality control systems are required for gamma radiation resistance in *C. neoformans*.

It is known that persistent exposure to gamma radiation induces change in the primary structure of the proteins and affects their secondary or tertiary structures, causing protein degradation (42). Our transcriptome data showed that expression levels of genes belonging to the molecular chaperone and proteasome system were induced in response to gamma radiation, in accordance with other previous studies (34, 43) (Table S3).

**FIG 4:**
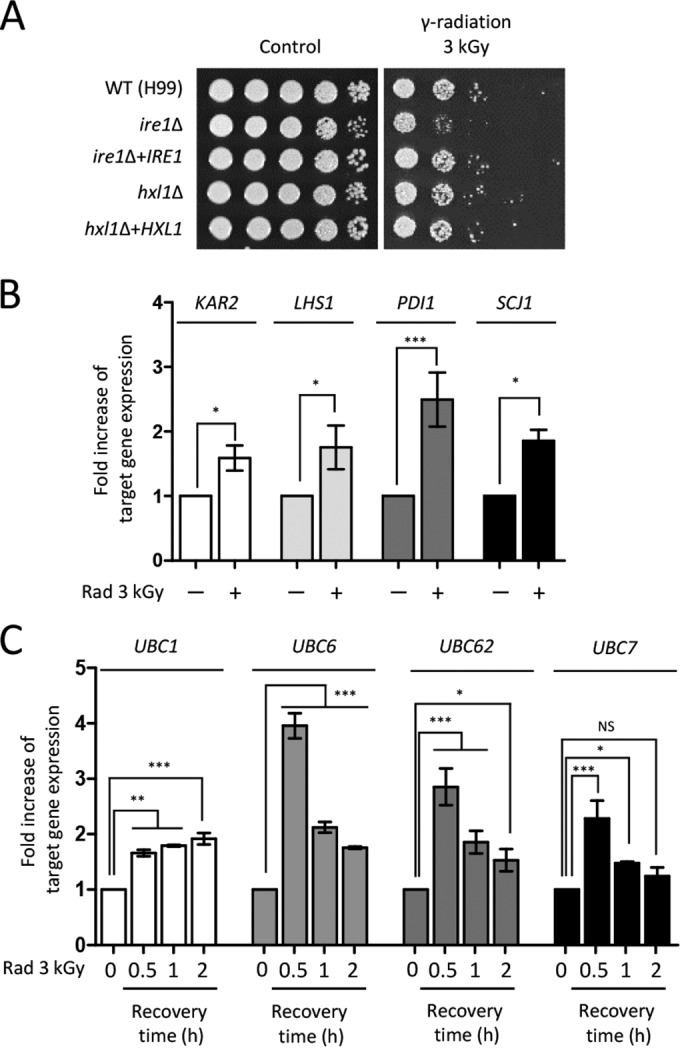
UPR and ERAD are involved in the gamma radiation resistance of *C. neoformans*. (A) The WT (H99), *ire1*Δ (YSB552), *ire1*Δ+*IRE1* (YSB1000), and *hxl1*Δ (YSB723) mutant, and *hxl1*Δ+*HXL1* complemented (YSB762) *C. neoformans* strains were grown overnight at 30°C in liquid YPD medium, and the 10-fold serially diluted cells were spotted onto solid YPD medium. Cells were exposed to gamma radiation and then further incubated at 30°C for 1 to 3 days. (B and C) Fold increase in expression of UPR downstream and ubiquitin-related genes after radiation exposure. The expression patterns of target genes were quantitatively determined using qRT analysis with the gene-specific primers listed in Table S2. To monitor the fold increase in expression of molecular chaperone genes, the cDNA was synthesized with total RNAs extracted from cells recovered 60 min after exposure to gamma radiation or not exposed to gamma radiation. Duplicate technical experiments with two or more biological samples were conducted. Representative images from independent experiments for each target gene are shown. Error bars indicate standard deviations. Asterisks indicate statistical significance of differences in expression levels of each gene (*, *P* < 0.05; **, *P* < 0.01; ***, *P* < 0.001). NS, not significant.

To investigate further the roles of protein quality control systems in the radiation response of *C. neoformans*, we first examined the unfolded protein response (UPR) pathway, which controls the expression levels of molecular chaperones in yeasts and humans (44). The *C. neoformans* UPR pathway consists of Ire1 kinase and its downstream transcription factor (TF), Hxl1 (45, 46). Here we found that the *ire1*Δ mutant, not the *hxl1*Δ mutant, exhibited growth defects when exposed to gamma radiation (Fig. 4A), suggesting that Ire1 was involved in radiation resistance in an Hxl1-independent manner. In addition, using qRT-PCR, we monitored expression levels of the molecular chaperones and genes required for protein folding, which are known to be regulated by the UPR pathway. Our results showed that the expression levels of *KAR2* (CNAG_06443; an endoplasmic reticulum [ER]-resident molecular chaperone), *LHS1* (CNAG_03899; a molecular chaperone of the heat shock protein 70 [HSP70] family), *PDI1* (CNAG_06240; a protein disulfide isomerase), and *SCJ1* (CNAG_05252; a homolog of *S. cerevisiae* DnaJ) were all increased in response to gamma radiation (Fig. 4B). All of these results indicated that the UPR pathway played a role in the gamma radiation resistance in *C. neoformans*.

In addition to the UPR pathway, the ubiquitin-mediated endoplasmic reticulum-associated degradation (ERAD) pathway plays critical roles in protein quality control of eukaryotic cells (47). To reveal the connection between the ERAD pathway and effects of gamma radiation, we measured the expression levels of ubiquitin enzymes belonging to the ERAD pathway. The expression levels of *UBC6*, *UBC62*, and *UBC7* genes were greatly increased at 30 min after gamma radiation exposure and then gradually decreased. In contrast, expression of *UBC1* gradually increased after radiation exposure (Fig. 4C). Taken together, protein quality control systems including the UPR and ERAD pathways contribute to radiation resistance by regulating the expression levels of genes of molecular chaperones and protein degradation.

### Ergosterol homeostasis is required for the gamma radiation resistance of *C. neoformans*.

**FIG 5:**
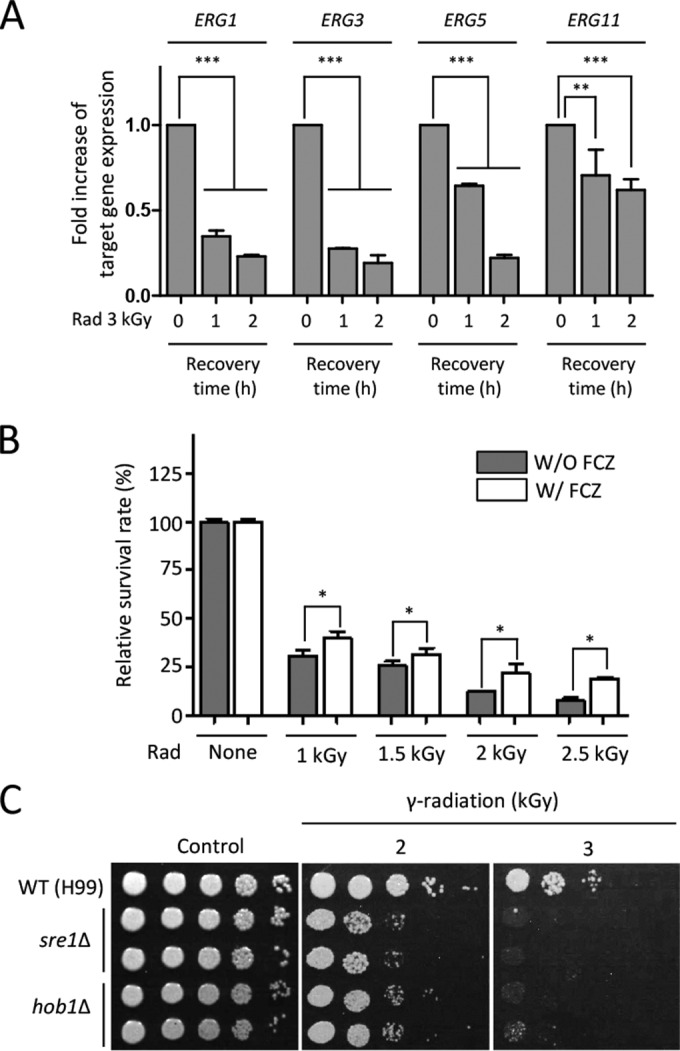
Homeostasis of ergosterol biosynthesis is required for the gamma radiation resistance of *C. neoformans*. (A) Expression analysis of *ERG* genes at the recovered time after exposure to radiation. The fold increase of *ERG* gene expression was quantitatively monitored using qRT analysis with the gene-specific primers listed in Table S2. Duplicate technical experiments with two biological samples were performed. Representative images from independent experiments for each *ERG* gene are shown. Error bars indicate standard deviations. Asterisks represent statistical significance of differences in expression levels of each *ERG* gene (**, *P* < 0.01; ***, *P* < 0.001). (B) Reduced ergosterol increased in the gamma radiation resistance in *C. neoformans*. *C. neoformans* WT (H99) strains were grown in a liquid medium at 30°C for 16 h, and then the grown cells were subcultured for 5 h to an OD_600_ of 1.0. Cells were treated with or without fluconazole (10 µg/ml) for 90 min. Next, cells treated with or without fluconazole were exposed to the indicated dose of gamma radiation for 1 h. After radiation, cells were spread onto the solid YPD medium and further incubated at 30°C for 2 days. The relative survival rate was measured as the viable cell count number at the indicated dose of radiation divided by the viable cell count number before radiation. The relative survival rates were statistically compared between cells treated with or without fluconazole using the Bonferroni selected comparison test performed with Prism software, version 5.0 (GraphPad Software, Inc.). Asterisks represent statistical significance of differences in the relative survival rate (*, *P* < 0.05). (C) Ergosterol homeostasis is required for gamma radiation resistance. *C. neoformans* WT (H99), *sre1*Δ (YSB2493 and YSB2494), and *hob1*Δ (YSB2308 and YSB2309) mutants grown overnight were 10-fold serially diluted (1 to 10^4^) and then spotted onto the YPD medium. Cells were exposed to the indicate dose of gamma radiation for 1 h and then further incubated at 30°C for 3 days.

Previous studies have revealed that the expression levels of genes involved in ergosterol biosynthesis were downregulated in response to genotoxic DNA damage agents, including MMS and gamma radiation in *S. cerevisiae* (34, 48). Our transcriptome analysis also revealed that expression levels of ergosterol biosynthesis and lipid metabolic genes were suppressed following exposure to gamma radiation (Table S3). In agreement with the DNA microarray data, the qRT-PCR analysis confirmed that *ERG1*, *ERG3*, *ERG5*, and *ERG11* were downregulated (Fig. 5A).

To demonstrate that a decreased level of cellular ergosterol biosynthesis enhanced resistance to gamma radiation, we artificially suppressed cellular ergosterol content by treatment with fluconazole, which is an inhibitor of Erg11 (lanosterol 14-α-demethylase), and then we tested whether gamma radiation resistance could be increased or not. Indeed, fluconazole treatment increased gamma radiation resistance in wild-type cells, and the effect was observed more clearly with high doses of radiation (Fig. 5B), suggesting that decreased ergosterol content contributed to gamma radiation resistance.

To support further that cellular ergosterol homeostasis was involved in gamma radiation resistance, we tested the gamma radiation resistance of strains with Sre1 or Hob1 deleted, which are 2 TFs affecting sterol biosynthesis in *C. neoformans* (49, 50). Both *sre1*Δ and *hob1*Δ mutants exhibited severe growth defects when exposed to gamma radiation (Fig. 5C). Notably, because Sre1 is the major positive regulator of expression of ergosterol biosynthesis genes, this finding indicated that homeostasis of ergosterol biosynthesis was critical for gamma radiation resistance in *C. neoformans*.

### Discovery of novel gamma radiation resistance genes in *C. neoformans*.

**FIG 6:**
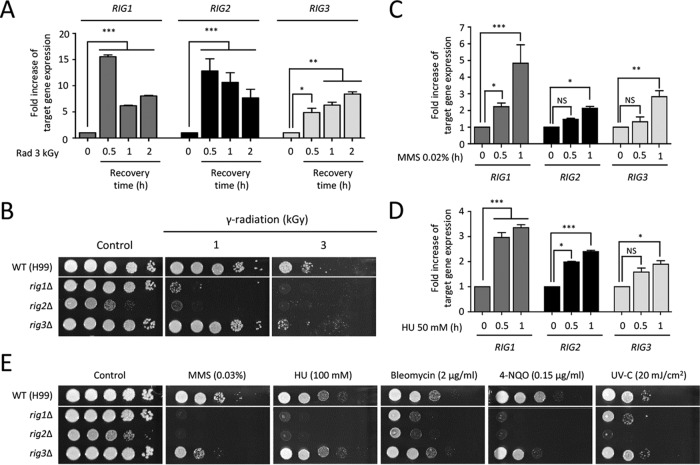
Identification of radiation-responsive genes in *C. neoformans*. (A, C, and D) Quantitative measurement of fold increase in expression of radiation-induced genes. The fold increase of the target gene expression was determined by qRT-PCR analysis with the gene-specific primers listed in Table S2. The cDNA was synthesized with total RNAs extracted from H99 strains recovered 30, 60, and 120 min after exposure to gamma radiation or not exposed to gamma radiation (A). The qRT analysis was performed with the cDNA synthesized from total RNA isolated from WT H99 strains grown in YPD medium containing 0.02% MMS (C) or 50 mM HU (D). Duplicate technical experiments with two independent biological samples were performed. Representative images from independent experiments for each radiation-responsive gene are displayed. Error bars indicate standard deviations. Asterisks indicate statistical significance of differences in the relative expression levels (*, *P* < 0.05; **, *P* < 0.01; ***, *P* < 0.001). NS, not significant. (B and E) *RIG1*, *RIG2*, and *RIG3* genes were mainly involved in gamma radiation resistance, as well as DNA damage response. Each *C. neoformans* strain (WT [H99] or *rig1*Δ [KW158], *rig2*Δ [KW160], or *rig3*Δ [KW96] mutant) was grown overnight at 30°C in liquid YPD medium, and 10-fold serially diluted cells (1 to 10^4^ dilutions) were spotted onto the YPD agar medium. Strains were exposed to the indicated dose of gamma radiation for 1 h. For the DNA damage test, 10-fold serially diluted cells were spotted onto YPD agar medium containing the indicated concentration of agents. The two images split by a horizontal white line in each spot assay were obtained from the same plate.

Among the radiation-responsive genes identified by our transcriptome analysis, we further characterized the functions of genes that exhibited differential expression patterns at statistically significantly levels (28 genes, >2-fold [*P* < 0.05]) during recovery from gamma radiation (Table S3) but do not belong to the environmental stress-regulated genes in *C. neoformans* (51). We characterized the roles of genes that are evolutionarily conserved in other fungi but the functions of which have not been studied in *C. neoformans*. These genes included CNAG_04055 (homologous to *Schizosaccharomyces pombe* Rad1, which is functionally homologous to *S. cerevisiae RAD17*), CNAG_03659 (homologous to *S. pombe* Rad4, which is functionally homologous to *S. cerevisiae DPB11*), and CNAG_03813 (homologous to *S. pombe* Ssb3, which is functionally homologous to *S. cerevisiae RFA3*). The qRT-PCR analysis confirmed that the expression patterns of these three genes were increased, verifying our DNA microarray data (Fig. 6A). Based on this finding, we designated these genes *RIG1* (radiation-induced gene 1; CNAG_04055), *RIG2* (CNAG_03659), and *RIG3* (CNAG_03813).

To address the roles of these genes in gamma radiation resistance, we constructed *rig1*Δ, *rig2*Δ, and *rig3*Δ mutants. The *rig1*Δ, *rig2*Δ, and *rig3*Δ mutants displayed growth defects when exposed to gamma radiation (Fig. 6B). Independently constructed strains for each mutant exhibited identical phenotypes (data not shown). The *rig2*Δ mutant showed severe growth retardation under basal conditions as well as radiation conditions, whereas the *rig1*Δ mutant exhibited growth defects only under radiation conditions. In contrast to the dramatic roles of Rig1 and Rig2, Rig3 appeared to play a minor role in radiation resistance because the *rig3*Δ mutant was susceptible only to a high dose of gamma radiation (3 kGy) (Fig. 6B).

Given that genes exhibiting functional identity to Rig1, Rig2, and Rig3 are involved in the DNA repair system in *S. cerevisiae*, we further investigated whether these proteins had functions in genotoxic stress responses as well as gamma radiation resistance. First, we monitored the expression patterns of these genes under treatment with HU or MMS using qRT-PCR analysis. The expression of *RIG1*, *RIG2*, and *RIG3* was gradually increased in response to either HU or MMS treatment (Fig. 6C and D).

Next, we observed the DNA damage resistance of strains with the *RIG* gene deleted. In agreement with the results of radiation exposure, both *rig1*Δ and *rig2*Δ mutants showed severe sensitivity to all of the DNA-damaging stresses that we tested (Fig. 6E). This result was in strong agreement with the expression data of *RIG1* and *RIG2*. The *rig3*Δ mutant exhibited increased susceptibility to MMS and 4-NQO. In conclusion, radiation-induced genes, such as *RIG1*, *RIG2*, and *RIG3*, contributed to gamma radiation resistance, as well as DNA damage stress responses, in *C. neoformans*.

### Identification and characterization of a novel bZIP TF, Bdr1, for radiation resistance in *C. neoformans*.

**FIG 7:**
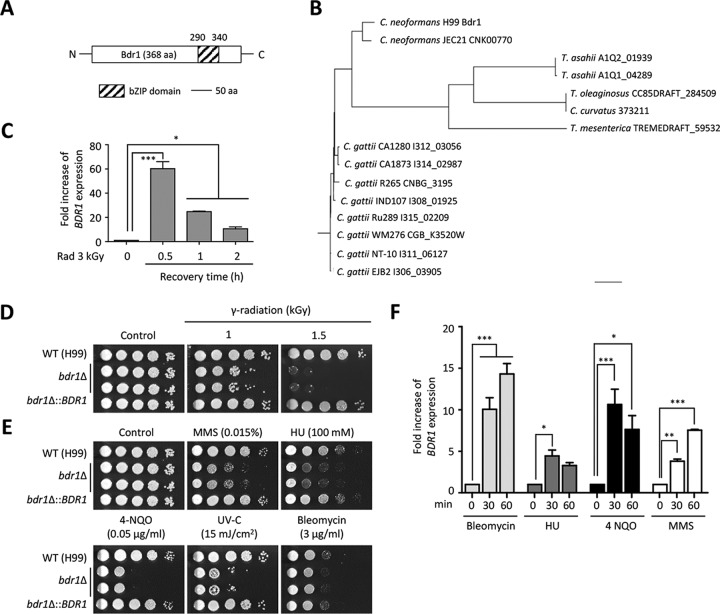
A novel TF, Bdr1, plays essential roles in both gamma radiation resistance and genotoxic DNA damage stress in *C. neoformans*. (A) The CNAG_02589 diagram shows the functional bZIP domain, which was identified by the Conserved Domain Search Service (CD Search; http://www.ncbi.nlm.nih.gov/Structure/cdd/wrpsb.cgi). (B) Phylogenetic tree of the Bdr1 homologs in fungi. The phylogenetic tree was generated by a philodendron phylogenetic tree printer (http://iubio.bio.indiana.edu/treeapp/treeprint-form.html). The scale bar represents an evolutionary distance of 0.1. (C and F) Fold increase of the *BDR1* gene expression post-radiation exposure (3 kGy) and under genotoxic stress (bleomycin [3 µg/ml], HU [50 mM], MMS [0.02%], and 4-NQO [0.15 µg/ml]). (D and E) Bdr1 is required for both gamma radiation resistance and DNA damage stress. The WT (H99), *bdr1*Δ mutants (KW137 and KW138), and *bdr1*Δ::*BDR1* complemented (KW193) *C. neoformans* strainswere grown overnight at 30°C in liquid YPD medium, and 10-fold serially diluted cells (1 to 10^4^ dilutions) were spotted onto the YPD agar medium containing DNA damage stress inducers. For the UV-C and gamma radiation resistance test, serially diluted cells were exposed to gamma radiation or UV-C and then further incubated at 30°C for 1 to 3 days. For the qRT analysis, experiments with two or more biological samples were performed. Representative images from independent experiments for each gene are shown. Error bars indicate standard deviations. Asterisks indicate statistical significance of differences in the relative expression levels (*, *P* < 0.05; **, *P* < 0.01; and ***, *P* < 0.001).

Although *C. neoformans RIG1*, *RIG2*, and *RIG3* functionally contribute to gamma radiation resistance, it still remains elusive how the fungus controls gamma radiation resistance, compared with radiation-sensitive fungi. To this end, we searched for *C. neoformans*-specific genes potentially involved in signal transduction, such as kinase, phosphatase, or TFs, among the genes induced post-radiation exposure. As a result, we identified CNAG_02589, which contains the bZIP domain (E value, 5.67e−07) in the C-terminal region, through the Conserved Domain Search Service (CD Search; http://www.ncbi.nlm.nih.gov/Structure/cdd/wrpsb.cgi) (Fig. 7A). Based on its dominant role in regulating a variety of DNA damage-responsive genes as described below, here we named this gene product Bdr1 (a bZIP TF for DNA damage response 1). Based on the phylogenetic analysis, Bdr1 is mainly found in the pathogenic *Cryptococcus* species and is evolutionarily distant from other fungal homologs (Fig. 7B)

To confirm radiation-dependent expression changes in *BDR1*, qRT-PCR analysis was performed. Expression of *BDR1* was strongly increased at 30 min post-radiation recovery and then gradually decreased (Fig. 7C). To demonstrate the role of Bdr1 in gamma radiation resistance, we constructed *bdr1*Δ mutant strains and performed survival testing. In accordance with the strong induction of *BDR1* expression, deletion of *BDR1* caused severe growth defects upon radiation exposure, whereas its complemented strain (*bdr1*Δ*::BDR1*) restored radiation resistance to wild-type levels (Fig. 7D).

To address whether Bdr1 played a role in genotoxic stress responses, as well as radiation resistance, we compared the growth of the *bdr1*Δ mutant to that of the wild-type strain when cells were exposed to genotoxic stress inducers. The *bdr1*Δ mutant displayed severe growth defects when treated with MMS, HU, 4-NQO, and UV-C, but it showed weak growth retardation upon exposure to bleomycin (Fig. 7E). Supporting this function in genotoxic stress response and adaptation, *BDR1* was strongly induced by the genotoxic stressors bleomycin, 4-NQO, HU, and MMS (Fig. 7F).

### Identification of downstream targets and upstream regulators of Bdr1 for radiation resistance in *C. neoformans*.

**FIG 8:**
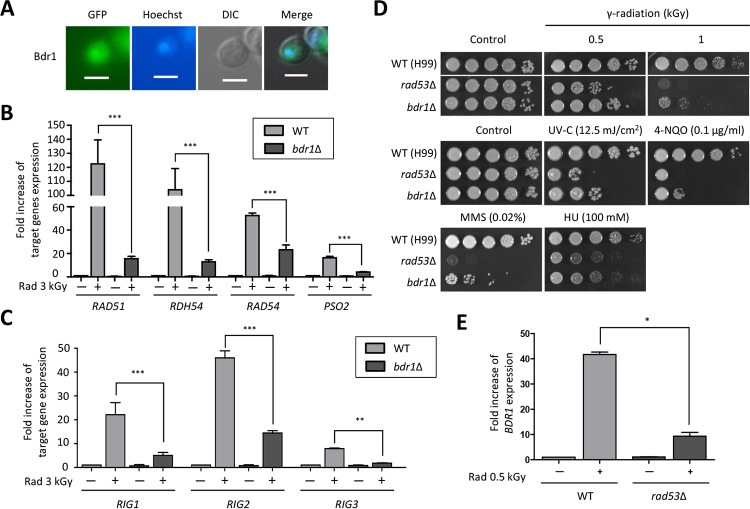
Identification of downstream and upstream factors of Bdr1 in *C. neoformans*. (A) Bdr1 is localized in the nucleus. The cellular localization of Bdr1 fused with GFP was visualized by fluorescence microscopy. Hoechst stain was used for nucleus staining after cells were fixed with formaldehyde. Scale bars represent 10 µm. (B and C) Bdr1 controls expression of DNA repair genes and *RIG* genes post-radiation exposure. (D) Rad53 is involved in both gamma radiation resistance and DNA damage stress. The two images split by a horizontal white line in each spot assay were obtained from the same plate. (E) Rad53 is an upstream factor of Bdr1 during the radiation exposure response. For qRT analysis, duplicate technical experiments with two biological samples were performed. Representative images from independent experiments for each gene are shown. Error bars indicate standard deviations. Asterisks indicate statistical significance of differences in the relative expression levels (*, *P* < 0.05; **, *P* < 0.01; and ***, *P* < 0.001).

To address whether Bdr1 is a TF, we monitored whether Bdr1 was localized in the nucleus. To this end, we constructed the *bdr1*Δ∷*BDR1*-*GFP* complemented strains by introducing a wild-type copy of the *BDR1* gene fused with the green fluorescent protein gene, *GFP*, and we monitored the cellular localization of Bdr1-Gfp in *C. neoformans*. We confirmed that Bdr1-Gfp was functional because the *BDR1-GFP* fusion gene restored the wild-type level of radiation resistance in the *bdr1*Δ strain (see Fig. S2C and S2D in the supplemental material). We found that Bdr1 was enriched in the nucleus under basal conditions, indicating that Bdr1 was likely to be a TF in *C. neoformans* (Fig. 8A).

Given that Bdr1 is indispensable for resistance to gamma radiation and other genotoxic agents, we hypothesized that Bdr1 could be a master regulator controlling the expression levels of genes involved in the DNA repair system. To determine this hypothesis, we compared the expression levels of genes contributing to gamma radiation resistance in the *bdr1*Δ mutant and wild-type strains. Notably, radiation-responsive induction of expression of *RAD51*, *RDH54*, *RAD54*, *PSO2*, *RIG1*, *RIG2*, and *RIG3* was considerably reduced in the *bdr1*Δ mutant compared to the wild type, suggesting that Bdr1 played critical roles in the regulating expression of DNA repair genes (Fig. 8B and C).

Because the expression levels of genes involved in the oxidative stress response, proteasome and molecular chaperone, and ergosterol biosynthesis post-gamma radiation exposure were changed, we determined whether Bdr1 regulated the expression levels of these genes after radiation exposure. In contrast to DNA repair genes, Bdr1 did not regulate expression of these genes (see Fig. S3 in the supplemental material). Supporting this finding, the *bdr1*Δ mutant exhibited wild-type levels of resistance in response to oxidative stress response, indicating that Bdr1 contributed to gamma radiation resistance controlling the expression of DNA repair genes (see Fig. S4A in the supplemental material).

The signal transduction mechanism that recognizes DNA damage is evolutionarily conserved from yeasts to humans (52). In particular, Rad53, which is orthologous to Chk2 in humans, plays critical roles in DNA damage responses and cell cycle regulation in *S. cerevisiae* (53). Recently, our study reported that perturbation of Rad53 checkpoint kinase in *C. neoformans* caused growth defects in response to genotoxic DNA damage stress (54). To demonstrate whether *C. neoformans* Rad53 was required for gamma radiation resistance, we compared the growth of the *rad53*Δ mutant to that of the wild-type strain in response to gamma radiation. The *rad53*Δ mutant showed severe growth defects after gamma radiation, as well as following exposure to genotoxic stress inducers, such as UV-C, 4-NQO, and bleomycin (Fig. 8D). Given that the *C. neoformans rad53*Δ mutant also exhibited susceptibility to gamma radiation exposure, as well as DNA damage insults, and *S. cerevisiae* Rad53 controls the transcriptional response of DNA damage-responsive genes through diverse TFs, we questioned whether Rad53 regulated the expression of *BDR1*. To answer this question, we measured the expression level of *BDR1* in wild-type and *rad53*Δ mutant strains post-radiation exposure. Notably, the increased *BDR1* gene expression in the wild-type strain postradiation was significantly decreased by deletion of *RAD53*, indicating that Rad53 was an upstream regulator of Bdr1 in *C. neoformans* (Fig. 8E).

Taken together, a unique TF, Bdr1, the expression of which was regulated by the evolutionarily conserved Rad53 protein kinase, was required for genotoxic stress as well as gamma radiation resistance controlling the expression of diverse DNA repair genes in *C. neoformans*.

## DISCUSSION

The goal of this study was to elucidate a genome-wide gamma radiation response mechanism in *C. neoformans*, which is known as a radiation-resistant eukaryotic microorganism found in highly radioactive environments (26). Our transcriptome analysis revealed that the expression levels of genes involved in DNA repair systems, molecular chaperones, and proteasomes were induced, while those involved in protein translation, metabolic process, and ergosterol synthesis were reduced in response to gamma radiation. The radiation-dependent transcriptome profile observed in *C. neoformans* was distinct from that of another melanized radiation-resistant fungus, *Wangiella dermatitidis* (55). A plethora of DNA repair genes were downregulated, and the ribosomal biogenesis, fatty acid, and lipid biosynthesis genes were upregulated in the irradiated *W. dermatitidis* (55). This difference might be attributable to the dose of radiation used. In this study, for the transcriptome analysis, *C. neoformans* was exposed to a lethal dose of gamma radiation (3 kGy for 1 h), but *W. dermatitidis* was irradiated with a chronic and low dose (14.4 mGy for 48 h) of gamma radiation (55). Considering the upregulation of DNA repair genes and the downregulation of ribosomal biogenesis genes in the model yeast *S. cerevisiae* exposed to a high dose of gamma radiation (0.17 kGy for 20 min), the dose of radiation and exposure time could have influenced the transcriptional repertoire of the organism (34). In addition, intrinsic melanin production ability might induce disagreement in these transcriptome results. Intrinsically melanized *W. dermatitidis* induces the expression of transporter and ribosomal biogenesis genes responding to radiation, but a nonmelanized *W. dermatitidis* mutant did not (55). The expression levels of genes related to transport and ribosomal biogenesis were decreased in nonintrinsically melanized *C. neoformans* (Table S3).

The common phenomenon observed in the transcriptome analyses of model yeasts and *W. dermatitidis* is that genes belonging to the oxidative stress response are upregulated (33, 34, 55). *S. cerevisiae* strains lacking cytosolic catalase exhibited increased susceptibility to IR (56, 57). In *C. neoformans*, however, we found that catalase was dispensable for radiation, but Srx1, which is required for the recycling of 2-Cys peroxiredoxin (Prx) (40), was required for gamma radiation resistance. However, *C. neoformans* 2-Cys Prx Tsa1 did not contribute to gamma radiation resistance, suggesting that Srx1 confers resistance in a 2-Cys Prx-independent manner. Srx1 plays a Prx-independent role in fungicide-dependent cell swelling and growth arrest (40). Srx1 also plays a role in the deglutathionylation of diverse proteins in response to oxidative stress, thereby affecting cell signaling during oxidative stress (58). The Mn/Fe ratio of *C. neoformans* (0.039) was not higher than that of *S. cerevisiae* (0.093). Considering the various ROS detoxification systems of *T. gammatolerans* and *P. furiosus* (14, 15), the Mn/Fe of which are comparable to those of IR-sensitive bacteria (8), it is likely that *C. neoformans* possesses additional antioxidative systems that can limit ROS production and/or nullify ROS toxicity.

The ability of IR-resistant organisms to minimize protein damage from IR-induced ROS allows them to retain cellular function (2). Supporting this notion, our study showed increased expression levels of genes involved in molecular chaperone activity for protein protection and in the ubiquitin system for degradation of damaged proteins. Furthermore, autophagy-activating genes (*ATG8*, *ATG3*, and *ATG4*) were increased during recovery postradiation (see Fig. S5A in the supplemental material), indicating that cells employ recycling of cellular organelles damaged by radiation through autophagy processes. Although deletion of *ATG8*, *ATG3*, and *ATG4* did not influence gamma radiation resistance (Fig. S5B), it is possible that these autophagy-related proteins might have redundant functions in radiation resistance. Given that autophagy is also activated in response to gamma irradiation in mammals (59), further experiments are needed to elucidate the relationship between autophagy and radiation resistance in *C. neoformans*.

Here, we demonstrated that suppression of ergosterol biosynthesis contributed to gamma radiation resistance. Nevertheless, sophisticated homeostasis of ergosterol biosynthesis appears to be more critical for stress resistance than simple downregulation of ergosterol biosynthesis. Supporting this finding, this study and previous studies showed that deletion of *SRE1*, which is a key positive regulator of ergosterol biosynthesis genes, increased susceptibility to oxidative stress (49) and gamma radiation. Furthermore, *S. cerevisiae erg3*Δ and *erg6*Δ mutants were also hypersensitive to hydrogen peroxide (60). Taken together, our data supported that membrane remodeling and homeostasis through modulation of ergosterol biosynthesis enabled cells to counteract stress responses caused by gamma radiation exposure.

We found that multiple pathways and cellular functions are involved in gamma radiation resistance of *C. neoformans*, indicating that the gamma radiation resistance mechanism could be complex in the fungus. This study revealed that the mechanism of gamma radiation resistance in *C. neoformans* appears to share features with those of both radiation-sensitive and -resistant organisms. First, the gene regulation network of *C. neoformans* is similar to those of *S. cerevisiae* and *S. pombe* in response to radiation. However, the roles of *C. neoformans* genes involved in the DNA repair system are distinct from those of *S. cerevisiae* and *S. pombe*. The DNA repair function of Rdh54 was enhanced in *C. neoformans* compared to *S. cerevisiae* (35). Dpb11 is an essential protein required for replisome assembly and for the DNA damage checkpoint in *S. cerevisiae* (61). However, Rig2, which is the functional Dpb11 ortholog in *C. neoformans*, is not essential for viability but is required for normal growth and the DNA damage response. Second, *C. neoformans* undergoes a biotrophic life cycle similar to those of other radiation-resistant fungi, such as *U. maydis* and *Alternaria alternata* (16). Third, melanin pigment is involved in radiation resistance of *C. neoformans* by conferring antioxidant activity to cells. Melanized *C. neoformans* exhibits better growth than nonmelanized *C. neoformans* when treated with radiation (27). Melanin also functions in energy transduction post-radiation exposure (27). Therefore, the radiation resistance mechanism of *C. neoformans* is complex and is likely to be influenced by multiple cellular factors.

A previous large-scale functional analysis of *C. neoformans* TFs uncovered several mutants exhibiting altered susceptibility to genotoxic stresses (49). However, most of them were involved in normal growth or played pleiotropic roles in diverse stress responses, in stark contrast to Bdr1, which plays genotoxic stress-specific roles. Bdr1 was not involved in virulence factor formation (melanin and capsule) or thermotolerance (Fig. S4B to E). Interestingly, Bdr1 is distinct from another genotoxic stress-related TF, Rfx1 (regulatory factor for X box), which is primarily involved in DNA damage responses and highly conserved from yeasts to humans (62–65), and is phylogenetically confined to the pathogenic *Cryptococcus* species complex. Nevertheless, its expression is regulated by the evolutionarily conserved Rad53 kinase. Rad53 belongs to the Chk cell cycle checkpoint kinase family and mainly governs DNA damage responses from yeasts to humans (66). A similar phenomenon has been also reported in the *C. neoformans* UPR pathway, in which the evolutionarily conserved Ire1 kinase regulates the evolutionarily divergent TF Hxl1 (46). Therefore, it does not seem uncommon that an evolutionarily divergent fungal TF is controlled by a conserved upstream kinase, as suggested previously (49).

At this point, the detailed gamma radiation resistance mechanism mediated by Bdr1 in the pathogenic *Cryptococcus* species complex remains elusive. The *C. neoformans* var. *neoformans* JEC21 strain also has the Bdr1 homolog like the H99 strain, but is as susceptible to radiation as the *C. albicans* SC5314 strain, which does not contain the Bdr1 ortholog. Therefore, Bdr1 expression *per se* may not be a necessary and sufficient factor for gamma radiation resistance in the pathogenic *Cryptococcus* species complex. To confirm this hypothesis, we tested whether the expression of *BDR1* is induced post-radiation exposure in the JEC21 strain. Furthermore, we constructed a constitutive *BDR1-*overexpressing strain in the H99 strain background. We found that *BDR1* was induced on radiation in the JEC21 strain similar to the H99 strain, and *BDR1* overexpression did not further increase gamma radiation resistance of the H99 strain (see Fig. S6A and D in the supplemental material), supporting our hypothesis. Notably, however, we found that some Bdr1 downstream genes were induced at different levels in the JEC21 strain compared to the H99 strain. Expression of *RAD54*, *RIG1*, *RIG2*, and *RIG3* in the JEC21 strain was induced upon radiation as they were expressed in the H99 strain, but the induction levels of *RAD51* and *RDH54* were much lower in the JEC21 strain than the H99 strain (Fig. S6E). These data present several possibilities. First, posttranscriptional or -translational modifications (e.g., phosphorylation) of Bdr1 could be different between the two strains. Second, serotype-specific proteins may collaborate with Bdr1 (e.g., as a coactivator), contributing to the gamma radiation resistance of serotype A H99. Finally, there is a possibility that DNA binding and/or transcription activation activity of Bdr1 might be different between the JEC21 and H99 strains. Supporting this, 3 amino acids were different between the bZIP domains of the H99 and JEC21 strains, which may affect the DNA binding activity of Bdr1 (Fig. S6F). To further address these possibilities, comparative transcriptome analysis (RNA-seq) of H99 and JEC21 strains under radiation exposure and subsequent comprehensive functional analyses of the differentially regulated genes will be needed in future studies, as well as structural analysis of Bdr1.

In conclusion, our study provides comprehensive insight into genome-wide gamma radiation resistance networks in radiation-resistant fungi as well as *C. neoformans*.

## MATERIALS AND METHODS

### Strains and growth conditions.

The *C. neoformans* strains and primers used in this study are described in Tables S1 and S2 in the supplemental material and were cultured in YPD (yeast extract-peptone-dextrose) medium. For the total RNA isolation used in the DNA microarray, the wild-type H99S strain was used. Three independent cultures of the wild-type strain were prepared as biological replicates for the DNA microarray.

### Total RNA isolation for microarray.

The wild-type H99 strain was grown in 50 ml of YPD medium at 30°C for 16 h. Then, 2.5 ml of overnight cell culture was inoculated into 50 ml of fresh YPD medium and further incubated at 30°C until it reached an approximate optical density at 600 nm (OD_600_) of 1.0. The cells were transferred into Falcon tubes and irradiated at 3 kGy for 1 h. After gamma irradiation, the cells were transferred into the flask and incubated at 30°C for the required recovery times (30, 60, and 120 min). After recovery, the cells were treated with ice-cold ethanol/phenol stop solution (5% water-saturated phenol [pH < 7.0] in ethanol) followed by centrifugation. Next, the supernatant was discarded, and the cells were immediately stored at −70°C.

For total RNA isolation, 1 ml of RiboEx solution (Geneall) was added to the cell pellet and resuspended without bubbling. It was allowed to incubate at room temperature for 5 min. The cells was poured into the chilled screw-cap tube containing 1 ml of zirconium beads (Biospec, no. 11079105Z). The cells were homogenized by bead beating 4 times at 6,000× for 30 s with intermittent cooling at −20°C. Next, the cells were treated with 200 µl of chloroform and mixed vigorously for 3 min. After mixing, the samples were kept at room temperature for 2 min and then were centrifuged at 14,000 rpm at 4°C for 15 min. The aqueous phase was collected into a fresh tube. For purification of the extracted total RNA, we used an RNeasy spin column (Qiagen), following the manufacturer’s protocol.

### cDNA synthesis, Cy5 labeling, and microarray hybridization.

The synthesis of target cRNA probes and hybridization were performed using Agilent’s low-input Quick Amp WT labeling kit (Agilent Technology) according to the manufacturer’s instructions. Briefly, each 100 ng of total RNA was mixed with WT primer mix and incubated at 65°C for 10 min in cDNA master mix (5× first-strand buffer, 0.1 M dithiothreitol [DTT], 10 mM deoxynucleoside triphosphate [dNTP] mix, RNase-Out, and Moloney murine leukemia virus reverse transcriptase [MMLV RT]) was prepared and added to the reaction mixture. The samples were incubated at 40°C for 2 h, and then the RT and double-stranded DNA (dsDNA) synthesis was terminated by incubation at 70°C for 10 min.

The transcription master mix was prepared per the manufacturer’s protocol (4× transcription buffer, 0.1 M DTT, NTP mix, 50% polyethylene glycol [PEG], RNase-Out, inorganic pyrophosphatase, T7 RNA polymerase, and cyanine 5-CTP). Transcription of dsDNA was performed by adding the transcription master mix to the dsDNA reaction samples and incubating them at 40°C for 2 h. Amplified and labeled cRNA was purified on an RNase minicolumn (Qiagen) according to the manufacturer’s protocol. Labeled cRNA target was quantified using an ND-1000 spectrophotometer (NanoDrop Technologies, Inc., Wilmington, DE).

After checking labeling efficiency, cyanine 5-labeled cRNA target was mixed, and the fragmentation of cRNA was performed by adding 10× blocking agent and 25× fragmentation buffer and incubating at 60°C for 30 min. The fragmented cRNA was resuspended with 2× hybridization buffer and directly pipetted onto assembled MYcroarray.com (*Cryptococcus_neoformans_*JEC21) 3X20K microarray. The arrays were hybridized at 57°C for 17 h using an Agilent hybridization oven (Agilent Technology). The hybridized microarrays were washed per the manufacturer’s washing protocol (Agilent Technology).

### Scanning and data analysis.

The hybridization images were analyzed by an Agilent DNA microarray scanner (Agilent Technology), and the data quantification was performed using Agilent Feature Extraction software 10.7 (Agilent Technology). The average fluorescence intensity for each spot was calculated, and the local background was subtracted. All data normalization and selection of fold-changed genes were performed using GenoWiz 4.0 (Ocimumbiosolutions, India). Genes were filtered by removing Flag-out genes in each experiment. Global normalization was performed. The averages of normalized ratios were calculated by dividing the average of normalized signal channel intensity by the average of normalized control channel intensity.

### Quantitative real-time RT-PCR.

To measure the relative expression of target genes, we performed quantitative RT-PCR (qRT-PCR) analysis with the target gene-specific primers listed in Table S2 using CFX Manager (Bio-Rad). The cDNA was synthesized using the PrimeScript first-strand cDNA synthesis kit (TaKaRa) with total RNAs extracted from irradiated cells. Relative expression levels of target genes were determined using the threshold cycle (2^−ΔΔ*CT*^) method. Statistical analyses were performed using Prism software version 5.0 (GraphPad Software, Inc.). Significant differences were determined using Bonferroni’s multiple-comparison test. We calculated the Pearson correlation coefficient (PCC) between the DNA microarray-based transcriptome analysis and qRT-PCR data using Prism 5.0 (GraphPad Software, Inc.).

### Construction of *Cryptococcus* mutant strains.

*Cryptococcus* genes were knocked out in the serotype A H99 strain background using double-joint PCR (DJ-PCR) strategies (67, 68). For the DJ-PCR method, primer pairs L1/L2 and R1/R2 were used to amplify the 5′- and 3′-flanking regions of the target genes, respectively, with H99 genomic DNA in the first round of PCR. The dominant selectable marker (NAT^r^) was amplified with M13Fe (M13 forward extended) and M13Re (M13 reverse extended) using pNAT. In the second round of PCR, target gene disruption cassettes with the 5′ or 3′ region of the NAT-split marker were amplified by DJ-PCR with the primer pair L1/B1455 or R2/B1454, respectively, using the first-round PCR product as a template. The split target gene disruption cassettes were introduced into the H99 strain by using the biolistic transformation method (69). Stable nourseothricin-resistant transformants were screened by diagnostic PCR with the primer set listed in Table S2. More than two independent mutants were constructed in the H99 background. The correct genotypes of positive transformants were validated by Southern blot analysis, as previously described (70) (see Fig. S2A and B and Fig. S7 in the supplemental material).

### Construction of *BDR1* complemented strains, Bdr1-Gfp strains, and *BDR1* overexpression strains.

To confirm the phenotypes observed in *bdr1*Δ mutants, complemented strains were generated as follows. The *BDR1* gene fragment containing its promoter, open reading frame (ORF), and terminator was PCR amplified using the primer pair J299/J300 harboring NotI restriction enzyme sites from the H99 genomic DNA. The amplified *BDR1* gene product was cloned into pGEM-T Easy (Promega), generating the plasmid pGEM-T-BDR1 (KWE12). After confirmed sequence errors, the NotI-digested *BDR1* gene insert was subcloned into the plasmid pJAF12 to produce the plasmid pJAF12-BDR1 (KWE16). The plasmid was linearized by restriction digestion with NsiI, which was in turn biolistically transformed into the *bdr1*Δ mutant (KW137). To demonstrate the targeted or ectopic integration of the *BDR1* gene, diagnostic PCR was executed.

To elucidate the cellular localization of Bdr1 in *C. neoformans*, the Bdr1-Gfp strain was constructed as follows. The promoter region of *BDR1* and the ORF of *GFP1* were amplified with the primer pairs J299/J320 and J321/J322, respectively. Next, these two PCR products were fused via PCR, using the primers J299 and J322. The overlap PCR product was cloned into pGEM-T Easy (Promega), producing the plasmid pGEM-T-BDR1PG (KWE29). The exon and terminator regions of *BDR1* were amplified with the primer pair J323/J319. Then the amplified PCR product was cloned into pGEM-T Easy (Promega), generating the plasmid pGEM-T-BDR1ET (KWE24). The two plasmids were sequenced to identify sequence errors. Next, the BamHI-digested pGEM-T-BDR1ET insert was subcloned into the plasmid pGEM-T-BDR1PG to generate the plasmid pGEM-T-BDR1PGET (KWE21). Then the NotI-digested pGEM-T-BDR1PGET insert was subcloned into the plasmid pJAF12 to produce plasmid pJAF12-BDR1PGET (KWE34). The NsiI-digested pJAF12-BDR1PGET was biolistically transformed into the *bdr1*Δ mutant (KW137). To verify the targeted or ectopic integration of the *BDR1* gene, diagnostic PCR was performed.

To construct *BDR1* overexpression strains, the histone H3 gene promoter with a selectable marker was inserted upstream of the ATG start codon of *BDR1* gene. First, the 5′-flanking region of *BDR1* was amplified with primers J312 and J313. The 5′-exon region of *BDR1* was amplified with primers J314 and J273. The *NEO-H3* promoter fragment was amplified with primers B4017 and B4018 from the plasmid pNEO-H3. The left fusion fragment was amplified with primers J312 and B1887 from templates containing the 5′-flanking region of *BDR1* and the *NEO-H3* promoter fragments. The right fusion fragment was amplified with primers J273 and B1886 from templates containing the 5′-exon region of *BDR1* and the *NEO-H3* promoter fragments. Then, the two DJ-PCR products were mixed and introduced into the serotype A H99 strain by biolistic transformation. We verified the correct insertion of the H3 promoter by Southern blot analysis and determined the basal expression levels of *BDR1* by qRT-PCR analysis (Fig. S6B and C).

### Gamma radiation and DNA damage stress tests.

Each *Cryptococcus* strain was cultured in liquid YPD medium at 30°C overnight, washed, and serially diluted (1 to 10^4^ dilutions) in distilled H_2_O. Cells were spotted onto solid YPD medium containing the indicated concentrations of DNA damage stress-inducing agents, including methyl methanesulfonate (MMS), hydroxyurea (HU), bleomycin, and 4-nitroquinoline *n*-oxide (4-NQO). For the gamma radiation resistance test, cells spotted onto the solid YPD medium were exposed to gamma radiation from ^60^Co. To monitor sensitivity to UV irradiation, spotted cells were exposed to UV irradiation between 100 and 300 J/m^2^ in a UV cross-linker (CX-2000; UVP, Inc.). The cells were incubated at 30°C for 1 to 3 days and photographed daily.

### Capsule and melanin assays.

For the capsule assay, each *Cryptococcus* strain was incubated at 30°C for 16 h in YPD medium, spotted onto agar-based Dulbecco’s Modified Eagle's (DME) medium, and further incubated at 37°C for 2 days. The cells were visualized by India ink staining. For the melanin assay, cultured cells were spotted on agar-based Niger seed medium and further incubated at 30 and 37°C for 3 days. The cells were photographed daily for 3 days.

### Accession number(s).

The microarray data generated by this study are available at Gene Expression Omnibus (GEO; https://www.ncbi.nlm.nih.gov/geo/query/acc.cgi?acc=GSE80230) under GenBank accession no. GSE80230.

## SUPPLEMENTAL MATERIAL

Figure S1  Change in expression levels of laccase genes (*LAC1* and *LAC2*) after radiation exposure. The fold increase of *LAC1* and *LAC2* expression was quantitatively measured using qRT analysis with the gene-specific primers listed in Table S2. To monitor expression levels of laccase genes, the cDNA was synthesized with total RNAs extracted from cells recovered for 0.5, 1, and 2 h after exposure to gamma radiation (1 kGy [A] or 3 kGy [B]) or not exposed to gamma radiation. Duplicate technical experiments with two biological samples were conducted. Representative images from independent experiments for each target gene are shown. Error bars indicate standard deviations. Asterisks indicate statistical significance of differences in expression levels of each gene (**, *P* < 0.01; ***, *P* < 0.001). NS, not significant. Download Figure S1, PDF file, 0.1 MB

Figure S2  Construction of *bdr1*Δ mutants. (A) Diagram for construction of the *bdr1*Δ mutants. (B) The correct genotype of each deletion mutant was verified by Southern blot analysis using genomic DNAs digested with the indicated restriction enzyme. Membrane was hybridized with *BDR1*-specific probes, washed, and developed. (C and D) Bdr1-Gfp1 is functional. Each *C. neoformans* strain (WT [H99], *bdr1*Δ mutant [KW137], or *bdr1*Δ∷*BDR1-GFP* complemented strain [KW219]) was grown overnight at 30°C in liquid YPD medium, and 10-fold serially diluted cells (1 to 10^4^ dilutions) were spotted onto the YPD agar medium. Strains were exposed to the indicated dose of gamma radiation for 1 h. For the DNA damage test, 10-fold serially diluted cells were spotted onto the YPD agar medium containing the indicated concentration of agents. The two images split by a horizontal white line in each spot assay were obtained from the same plate (C). Download Figure S2, PDF file, 0.1 MB

Figure S3  Bdr1 does not control expression of genes involved in oxidative stress, the molecular chaperone, the proteasome system, and ergosterol biosynthesis. Total RNA was isolated from recovered cells (30, 60, and 120 min) post-gamma radiation exposure (3 kGy for 1 h). The qRT-PCR analysis was performed with gene-specific primer listed in Table S2 using cDNA synthesized from the total RNA (for 30 min [A and C], 60 min [B], or 120 min [D]). Duplicate technical experiments with two biological samples were perfromed. Representative images from independent experiments for each gene were shown. Error bars indicated standard deviations. Asterisks indicate statistical significance of differences in the fold change of target gene expression (*, *P* < 0.05; **, *P* < 0.01; and ***, *P* < 0.001). Download Figure S3, PDF file, 0.1 MB

Figure S4  Bdr1 is not involved in oxidative stress, thermotolerance, or melanin and capsule production (A and B). Each *C. neoformans* strain (WT [H99], *bdr1*Δ mutant [KW137], or *bdr1*Δ::*BDR1* complemented strain [KW193]) was cultured overnight at 30°C in liquid YPD medium, and 10-fold serially diluted cells (1 to 10^4^ dilutions) were spotted onto the YPD agar medium containing the indicated concentration of oxidative stress inducers. For the thermotolerance test, a plate was incubated at 37°C. (C and D) For the melanin production assay, cells were spotted and grown on Niger seed medium (0.1% glucose) at 30°C for 3 days. For the capsule production assay, *C. neoformans* strains were spotted and grown on Dulbecco’s Modified Eagle's (DME) medium at 37°C for 2 days. Cells were scraped, resuspended in PBS-water, and visualized by India ink staining. Size bars indicate 10 µm. (E) The relative capsule volume was measured by calculating the ratio of the length of the packed cell volume phase per length of the total volume phase. Statistical difference in relative capsule size between strains was determined by Bonferroni’s multiple comparison test. NS, not significant. Download Figure S4, PDF file, 0.2 MB

Figure S5  The autophagy system is activated in response to gamma radiation. (A) Expression patterns of autophagy related to genes. The fold increase of *ATG8*, *ATG3*, and *ATG4* gene expression was determined by using qRT-PCR analysis with each gene-specific primer. The cDNA was synthesized with total RNAs extracted from H99 strains recovered 0.5, 1, and 2 h after exposure to gamma radiation or not exposed to gamma radiation. Error bars indicate standard deviations. Asterisks indicate statistical significance of differences in expression levels of each gene (*, *P* < 0.05; **, *P* < 0.01; ***, *P* < 0.001). NS, not significant. (B) Atg8, Atg3, and Atg4 were not required for gamma radiation resistance in *C. neoformans*. *Cryptococcus* strains were cultured in the liquid YPD medium at 30°C overnight. Cells were serially diluted 10-fold (1 to 10^4^) and then spotted onto the YPD medium. Strains were exposed to the indicated dose of gamma radiation and then further incubated at 30°C for 3 days. Download Figure S5, PDF file, 0.1 MB

Figure S6  *BDR1* expression patterns and the effect of *BDR1* overexpression on the gamma radiation resistance of *Cryptococcus neoformans*. (A and E) Quantitative measurement of fold change of gene expression for *BDR1* (A) or radiation-induced genes (E) in the serotype A strain H99 and serotype D strain JEC21. The qRT-PCR analysis was performed with the gene-specific primers listed in Table S2 using cDNA synthesized from total RNA under basal conditions (0 h) or post-radiation recovery (0.5, 1, and 2 h for panel A and 0.5 h for panel E). Representative data from two independent experiments with duplicate measurement are exhibited. Error bars indicate standard deviations. Asterisks indicate statistical significance of differences in the fold change of gene expression (*, *P* < 0.05; **, *P* < 0.01; and ***, *P* < 0.001). (B) Construction of constitutive *BDR1-*overexpressing (*BRD1oe*) strain. (C) qRT-PCR analysis of *BDR1* expression in the *BDR1* overexpression strain. (D) The gamma radiation survival assay using the *BDR1* overexpression strain. Each *Cryptococcus* strain was cultured in the liquid YPD medium at 30°C overnight, serially diluted 10-fold (1 to 10^4^), and spotted onto the YPD medium. Cells were exposed to the indicated dose of gamma radiation and further incubated at 30°C for 2 days. (F) Sequence alignment of the bZIP domains of H99 and JEC21 Bdr1 proteins. Red boxes indicate nonidentical amino acids between the H99 and JEC21 Bdr1 proteins. Download Figure S6, PDF file, 0.1 MB

Figure S7  Construction of DNA damage repair, catalase, *RIG*, and autophagy gene mutants. The diagram for construction of each indicated mutant is shown in the left panel. The correct genotype of each deletion mutant was verified by Southern blot analysis using genomic DNAs digested with the indicated restriction enzyme (right panel). Each membrane was hybridized with each gene-specific probe, washed, and developed. Strains marked with asterisks were used in this study. Download Figure S7, PDF file, 0.3 MB

Table S1  Strains used in this study. Table S1, PDF file, 0.2 MB

Table S2  Primers used in this study. Table S2, PDF file, 0.1 MB

Table S3  The whole microarray data generated by this study. Table S3, XLSX file, 2.1 MB
