# Supplementary figures and images for: Unraveling Fungal Radiation Resistance Regulatory Networks through the Genome-Wide Transcriptome and Genetic Analyses of Cryptococcus neoformans
Source: mBio. 2016 Nov 29;7(6):e01483-16. doi: 10.1128/mBio.01483-16 (PMC5137497; doi:10.1128/mBio.01483-16)

**A**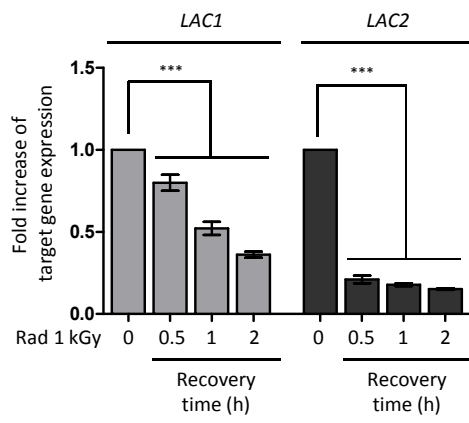**B**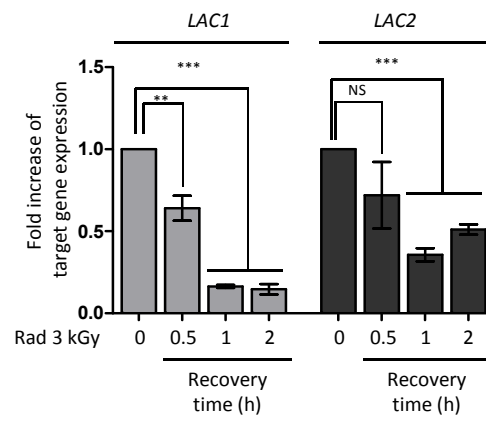

Supplement: Figure S1 — Change in expression levels of laccase genes (LAC1 and LAC2) after radiation exposure. The fold increase of LAC1 and LAC2 expression was quantitatively measured using qRT analysis with the gene-specific primers listed in Table S2. To monitor expression levels of laccase genes, the cDNA was synthesized with total RNAs extracted from cells recovered for 0.5, 1, and 2 h after exposure to gamma radiation (1 kGy [A] or 3 kGy [B]) or not exposed to gamma radiation. Duplicate technical experiments with two biological samples were conducted. Representative images from independent experiments for each target gene are shown. Error bars indicate standard deviations. Asterisks indicate statistical significance of differences in expression levels of each gene (**, P < 0.01; ***, P < 0.001). NS, not significant. Download [file mbo006163087sf1.pdf]

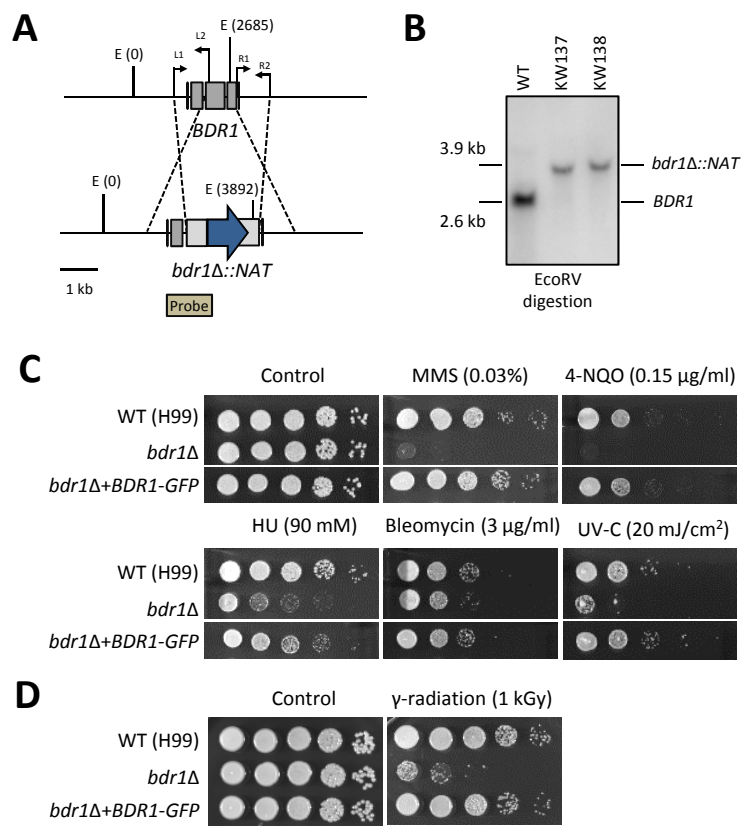

Supplement: Figure S2 — Construction of bdr1Δ mutants. (A) Diagram for construction of the bdr1Δ mutants. (B) The correct genotype of each deletion mutant was verified by Southern blot analysis using genomic DNAs digested with the indicated restriction enzyme. Membrane was hybridized with BDR1-specific probes, washed, and developed. (C and D) Bdr1-Gfp1 is functional. Each C. neoformans strain (WT [H99], bdr1Δ mutant [KW137], or bdr1Δ∷BDR1-GFP complemented strain [KW219]) was grown overnight at 30°C in liquid YPD medium, and 10-fold serially diluted cells (1 to 104 dilutions) were spotted onto the YPD agar medium. Strains were exposed to the indicated dose of gamma radiation for 1 h. For the DNA damage test, 10-fold serially diluted cells were spotted onto the YPD agar medium containing the indicated concentration of agents. The two images split by a horizontal white line in each spot assay were obtained from the same plate (C). Download [file mbo006163087sf2.pdf]

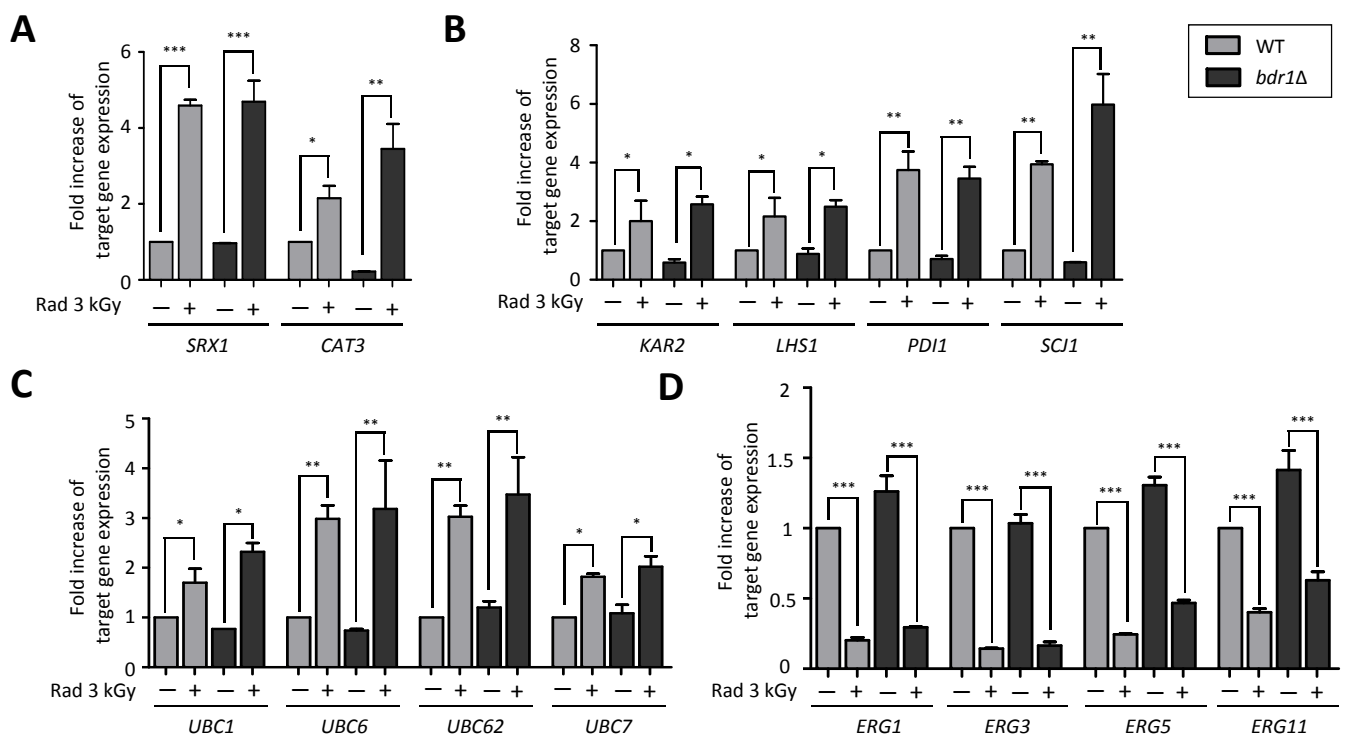

Supplement: Figure S3 — Bdr1 does not control expression of genes involved in oxidative stress, the molecular chaperone, the proteasome system, and ergosterol biosynthesis. Total RNA was isolated from recovered cells (30, 60, and 120 min) post-gamma radiation exposure (3 kGy for 1 h). The qRT-PCR analysis was performed with gene-specific primer listed in Table S2 using cDNA synthesized from the total RNA (for 30 min [A and C], 60 min [B], or 120 min [D]). Duplicate technical experiments with two biological samples were perfromed. Representative images from independent experiments for each gene were shown. Error bars indicated standard deviations. Asterisks indicate statistical significance of differences in the fold change of target gene expression (*, P < 0.05; **, P < 0.01; and ***, P < 0.001). Download [file mbo006163087sf3.pdf]

**A**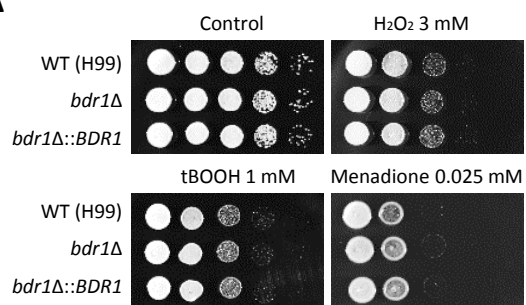**B**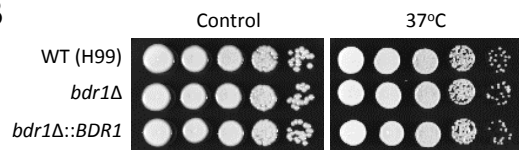**C**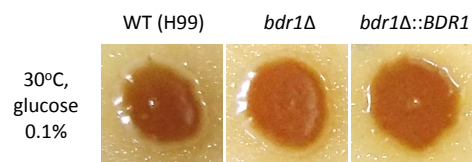**D**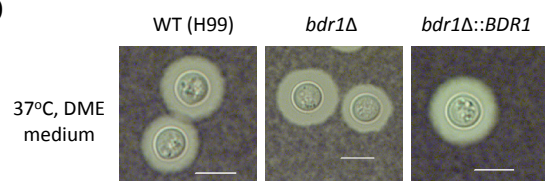**E**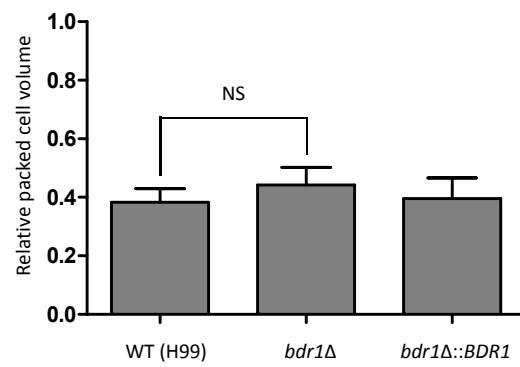

Supplement: Figure S4 — Bdr1 is not involved in oxidative stress, thermotolerance, or melanin and capsule production (A and B). Each C. neoformans strain (WT [H99], bdr1Δ mutant [KW137], or bdr1Δ::BDR1 complemented strain [KW193]) was cultured overnight at 30°C in liquid YPD medium, and 10-fold serially diluted cells (1 to 104 dilutions) were spotted onto the YPD agar medium containing the indicated concentration of oxidative stress inducers. For the thermotolerance test, a plate was incubated at 37°C. (C and D) For the melanin production assay, cells were spotted and grown on Niger seed medium (0.1% glucose) at 30°C for 3 days. For the capsule production assay, C. neoformans strains were spotted and grown on Dulbecco’s Modified Eagle's (DME) medium at 37°C for 2 days. Cells were scraped, resuspended in PBS-water, and visualized by India ink staining. Size bars indicate 10 µm. (E) The relative capsule volume was measured by calculating the ratio of the length of the packed cell volume phase per length of the total volume phase. Statistical difference in relative capsule size between strains was determined by Bonferroni’s multiple comparison test. NS, not significant. Download [file mbo006163087sf4.pdf]

**A**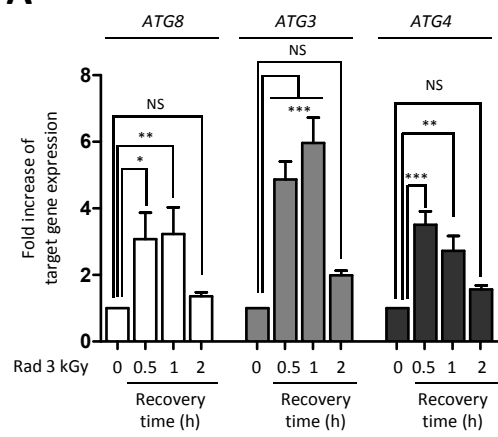**B**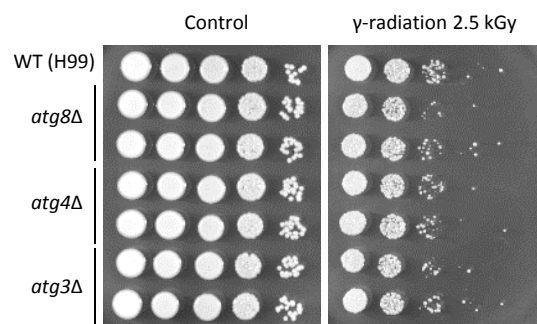

Supplement: Figure S5 — The autophagy system is activated in response to gamma radiation. (A) Expression patterns of autophagy related to genes. The fold increase of ATG8, ATG3, and ATG4 gene expression was determined by using qRT-PCR analysis with each gene-specific primer. The cDNA was synthesized with total RNAs extracted from H99 strains recovered 0.5, 1, and 2 h after exposure to gamma radiation or not exposed to gamma radiation. Error bars indicate standard deviations. Asterisks indicate statistical significance of differences in expression levels of each gene (*, P < 0.05; **, P < 0.01; ***, P < 0.001). NS, not significant. (B) Atg8, Atg3, and Atg4 were not required for gamma radiation resistance in C. neoformans. Cryptococcus strains were cultured in the liquid YPD medium at 30°C overnight. Cells were serially diluted 10-fold (1 to 104) and then spotted onto the YPD medium. Strains were exposed to the indicated dose of gamma radiation and then further incubated at 30°C for 3 days. Download [file mbo006163087sf5.pdf]

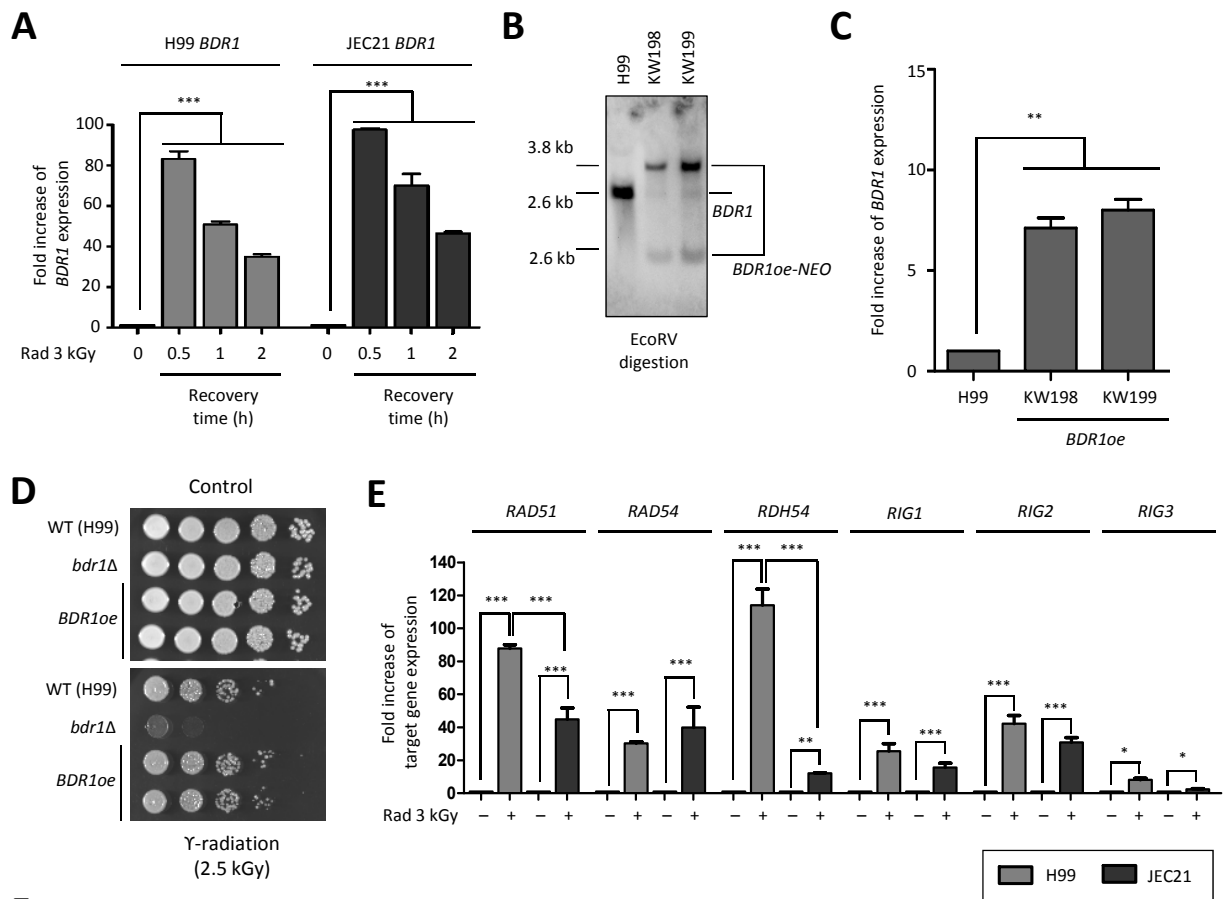

Supplement: Figure S6 — BDR1 expression patterns and the effect of BDR1 overexpression on the gamma radiation resistance of Cryptococcus neoformans. (A and E) Quantitative measurement of fold change of gene expression for BDR1 (A) or radiation-induced genes (E) in the serotype A strain H99 and serotype D strain JEC21. The qRT-PCR analysis was performed with the gene-specific primers listed in Table S2 using cDNA synthesized from total RNA under basal conditions (0 h) or post-radiation recovery (0.5, 1, and 2 h for panel A and 0.5 h for panel E). Representative data from two independent experiments with duplicate measurement are exhibited. Error bars indicate standard deviations. Asterisks indicate statistical significance of differences in the fold change of gene expression (*, P < 0.05; **, P < 0.01; and ***, P < 0.001). (B) Construction of constitutive BDR1-overexpressing (BRD1oe) strain. (C) qRT-PCR analysis of BDR1 expression in the BDR1 overexpression strain. (D) The gamma radiation survival assay using the BDR1 overexpression strain. Each Cryptococcus strain was cultured in the liquid YPD medium at 30°C overnight, serially diluted 10-fold (1 to 104), and spotted onto the YPD medium. Cells were exposed to the indicated dose of gamma radiation and further incubated at 30°C for 2 days. (F) Sequence alignment of the bZIP domains of H99 and JEC21 Bdr1 proteins. Red boxes indicate nonidentical amino acids between the H99 and JEC21 Bdr1 proteins. Download [file mbo006163087sf6.pdf]

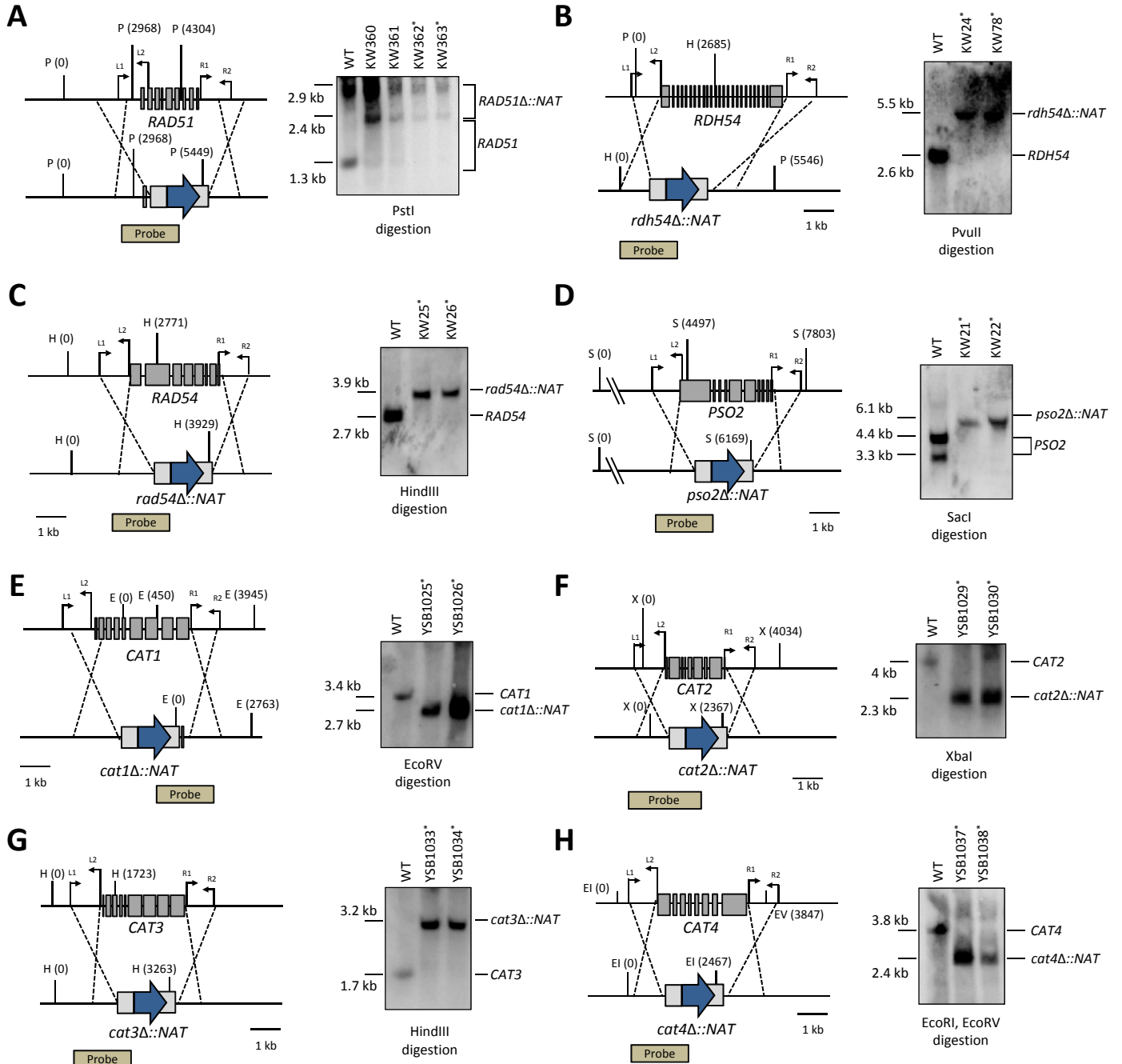

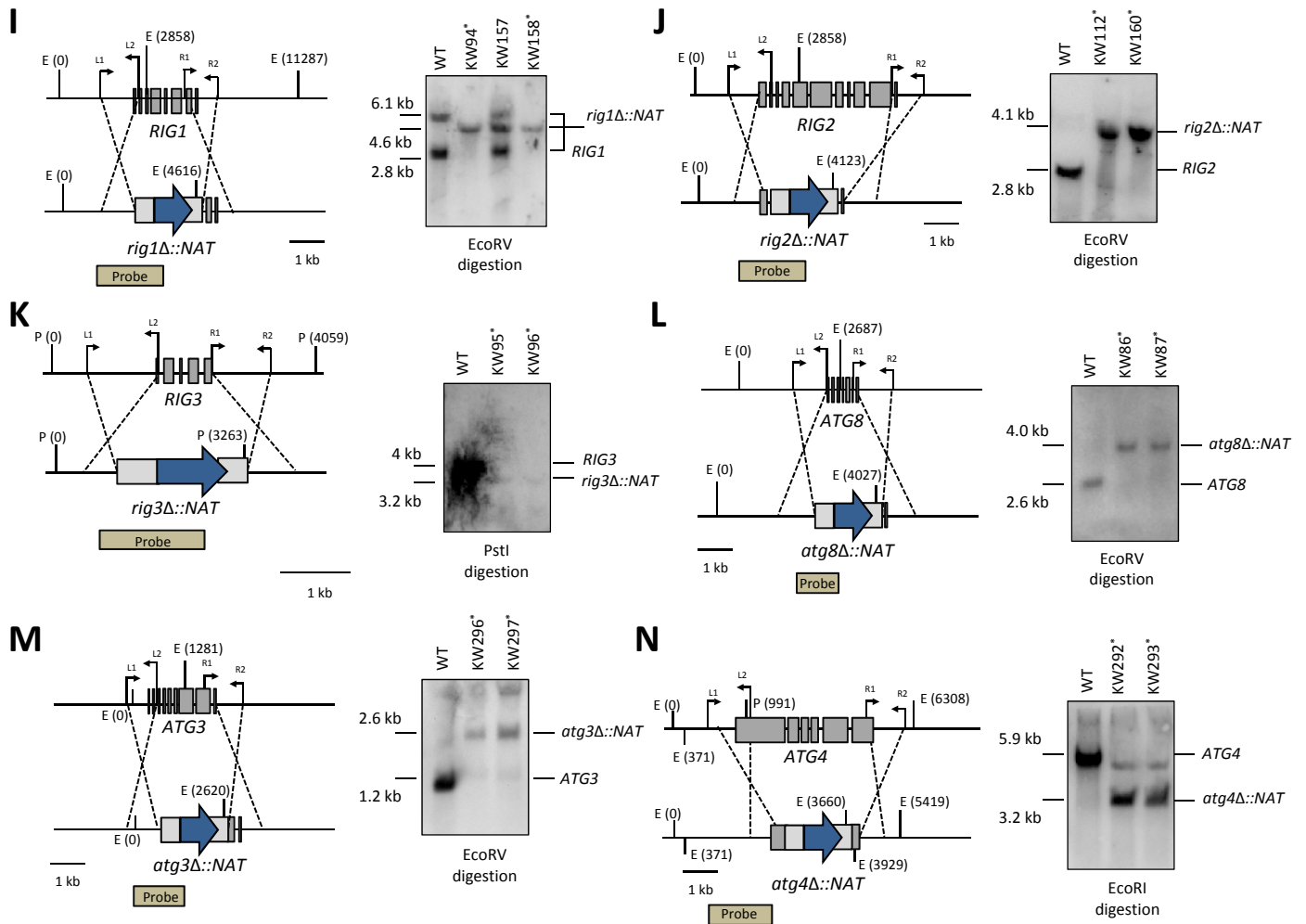

Supplement: Figure S7 — Construction of DNA damage repair, catalase, RIG, and autophagy gene mutants. The diagram for construction of each indicated mutant is shown in the left panel. The correct genotype of each deletion mutant was verified by Southern blot analysis using genomic DNAs digested with the indicated restriction enzyme (right panel). Each membrane was hybridized with each gene-specific probe, washed, and developed. Strains marked with asterisks were used in this study. Download [file mbo006163087sf7.pdf]
