# Supplementary material for: Unraveling Fungal Radiation Resistance Regulatory Networks through the Genome-Wide Transcriptome and Genetic Analyses of Cryptococcus neoformans
Source: mBio. 2016 Nov 29;7(6):e01483-16. doi: 10.1128/mBio.01483-16 (PMC5137497; doi:10.1128/mBio.01483-16)
Supplement: Table S1 — Strains used in this study. [file mbo006163087st1.pdf]

Table S1. Strains used in this study

Strain	Genotype	Parent	Reference
BY4742	*S. cerevisiae MATα his3Δ1 leu2Δ0 lys2Δ0 ura3Δ0*	S288C	(1)
SC5314	*C. albicans* wild-type		(2)
BG2	*C. glabrata* clinical isolate		(3)
H99	*MATα* serotype A		(4)
JEC21	*MATα* serotype D		(5)
R265	*MATα* VGIIa		(6)
WM276	*MATα* VGI		(6)
YSB552	*MATα ire1Δ::NAT-STM#224*	H99	(7)
YSB1000	*MATα ire1Δ::NAT-STM#224 IRE1-NEO*	YSB552	(7)
YSB723	*MATα hxl1Δ::NAT-STM#295*	H99	(7)
YSB762	*MATα hxl1Δ::NAT-STM#295 HXL1-NEO*	YSB723	(7)
YSB2493	*MATα sre1Δ::NAT-STM#240*	H99	(8)
YSB2308	*MATα hob1Δ::NAT-STM#213*	H99	(8)
YSB596	*MATα srx1Δ::NAT-STM#125*	H99	(9)
YSB1214	*MATα srx1Δ::NAT-STM#125 SRX1-NEO*	YSB596	(9)
YSB1273	*MATα tsa1Δ::NEO*	H99	(9)
YSB1204	*MATα tsa3Δ::NEO*	H99	(9)
YSB2735	*MATα tsa1Δ::NAT-STM#169 tsa3Δ::NEO*	YSB1204	(9)
YSB1667	*MATα trx1Δ::NAT-STM#234*	H99	(9)
YSB1791	*MATα trx2Δ::NEO*	H99	(9)
YSB1796	*MATα trx1Δ::NAT-STM#234 trx2Δ::NEO*	YSB1667	(9)
YSB3785	*MATα rad53Δ::NAT-STM#184*	H99	(10)
SG50	*MATα cat1::URA5 ura5*	H99	(11)
SG51	*MATα cat2::NAT*	H99	(11)
SG52	*MATα cat3::URA5 ura5*	H99	(11)
SG53	*MATα cat4::URA5 ura5*	H99	(11)
SG61	*MATα cat1::URA5 cat2::NAT cat3::URA5 ura5 cat3::URA5 cat4::NEO ura5*	SG60	(11)
KW24	*MATα rdh54Δ::NAT-STM#234*	H99	This study
KW78	*MATα rdh54Δ::NAT-STM#234*	H99	This study
KW25	*MATα rad54Δ::NAT-STM#234*	H99	This study
KW26	*MATα rad54Δ::NAT-STM#234*	H99	This study
KW362	*MATα rad51Δ::NAT-STM#56*	H99	This study
KW363	*MATα rad51Δ::NAT-STM#56*	H99	This study
KW21	*MATα pso2Δ::NAT-STM#234*	H99	This study
KW22	*MATα pso2Δ::NAT-STM#234*	H99	This study
KW94	*MATα rig1Δ::NAT-STM#230*	H99	This study
KW158	*MATα rig1Δ::NAT-STM#230*	H99	This study
KW112	*MATα rig2Δ::NAT-STM#234*	H99	This study
KW160	*MATα rig2Δ::NAT-STM#234*	H99	This study
KW95	*MATα rig3Δ::NAT-STM#230*	H99	This study
KW96	*MATα rig3Δ::NAT-STM#230*	H99	This study
KW137	*MATα bdr1Δ::NAT-STM#230*	H99	This study
KW138	*MATα bdr1Δ::NAT-STM#230*	H99	This study
KW193	*MATα bdr1Δ::NAT-STM#230 BDR1-NEO*	KW137	This study
KW219	*MATα bdr1Δ::NAT-STM#230 BDR1:GFP-NEO*	KW137	This study
KW86	*MATα atg8Δ::NAT-STM#5*	H99	This study
KW87	*MATα atg8Δ::NAT-STM#5*	H99	This study
KW296	*MATα atg3Δ::NAT-STM#230*	H99	This study
KW297	*MATα atg3Δ::NAT-STM#230*	H99	This study
KW292	*MATα atg4Δ::NAT-STM#230*	H99	This study
KW293	*MATα atg4Δ::NAT-STM#230*	H99	This study
YSB1025	*MATα cat1Δ::NAT-STM#177*	H99	This study
YSB1026	*MATα cat1Δ::NAT-STM#177*	H99	This study
YSB1029	*MATα cat2Δ::NAT-STM#288*	H99	This study
YSB1030	*MATα cat2Δ::NAT-STM#288*	H99	This study

YSB1033	*MATα cat3Δ::NAT-STM#169*	H99	This study
YSB1034	*MATα cat3Δ::NAT-STM#169*	H99	This study
YSB1037	*MATα cat4Δ::NAT-STM#146*	H99	This study
YSB1038	*MATα cat4Δ::NAT-STM#146*	H99	This study
KW198	*MATα* P*H3:BDR1-NEO*	H99	This study
KW199	*MATα* P*H3:BDR1-NEO*	H99	This study

Each *NAT-STM#* indicates the Nat^r marker with a unique signature tag.

References

1. **Brachmann CB, Davies A, Cost GJ, Caputo E, Li J, Hieter P, Boeke JD.** 1998. Designer deletion strains derived from *Saccharomyces cerevisiae* S288C: a useful set of strains and plasmids for PCR-mediated gene disruption and other applications. Yeast **14:**115-132.

2. **Gillum AM, Tsay EY, Kirsch DR.** 1984. Isolation of the *Candida albicans* gene for orotidine-5'-phosphate decarboxylase by complementation of *S. cerevisiae ura3* and *E. coli pyrF* mutations. Mol Gen Genet **198:**179-182.

3. **Cormack BP, Falkow S.** 1999. Efficient homologous and illegitimate recombination in the opportunistic yeast pathogen *Candida glabrata*. Genetics **151:**979-987.

4. **Perfect JR, Ketabchi N, Cox GM, Ingram CW, Beiser CL.** 1993. Karyotyping of *Cryptococcus neoformans* as an epidemiological tool. J Clin Microbiol **31:**3305-3309.

5. **Moore TD, Edman JC.** 1993. The α-mating type locus of *Cryptococcus neoformans* contains a peptide pheromone gene. Mol Cell Biol **13:**1962-1970.

6. **Kidd SE, Hagen F, Tscharke RL, Huynh M, Bartlett KH, Fyfe M, Macdougall L, Boekhout T, Kwon-Chung KJ, Meyer W.** 2004. A rare genotype of *Cryptococcus gattii* caused the cryptococcosis outbreak on Vancouver Island (British Columbia, Canada). Proc Natl Acad Sci USA **101:**17258-17263.

7. **Cheon SA, Jung KW, Chen YL, Heitman J, Bahn YS, Kang HA.** 2011. Unique evolution of the UPR pathway with a novel bZIP transcription factor, Hxl1, for controlling pathogenicity of *Cryptococcus neoformans*. PLoS Pathog **7:**e1002177.

8. **Jung KW, Yang DH, Maeng S, Lee KT, So YS, Hong J, Choi J, Byun HJ, Kim H, Bang S, Song MH, Lee JW, Kim MS, Kim SY, Ji JH, Park G, Kwon H, Cha S, Meyers GL, Wang LL, Jang J, Janbon G, Adedoyin G, Kim T, Averette AK, Heitman J, Cheong E, Lee YH, Lee YW, Bahn YS.** 2015. Systematic functional profiling of transcription factor networks in *Cryptococcus neoformans*. Nat Commun **6:**6757.

9. **Upadhya R, Kim H, Jung KW, Park G, Lam W, Lodge JK, Bahn YS.** 2013. Sulphiredoxin plays peroxiredoxin-dependent and -independent roles via the HOG signalling pathway in *Cryptococcus neoformans* and contributes to fungal virulence. Mol Microbiol **90:**630-648.

10. **Lee KT, So YS, Yang DH, Jung KW, Choi J, Lee DG, Kwon H, Jang J, Wang LL, Cha S, Meyers GL, Jeong E, Jin JH, Lee Y, Hong J, Bang S, Ji JH, Park G, Byun HJ, Park S, Park YM, Adedoyin G, Kim T, Averette AK, Choi JS, Heitman J, Cheong E, Lee YH, Bahn YS.** 2016. Systematic fungal analysis of kinases in the fungal pathogen *Cryptococcus neoformans*. Nat Commun **7:**12766.

11. **Giles SS, Stajich JE, Nichols C, Gerrald QD, Alspaugh JA, Dietrich F, Perfect JR.** 2006. The *Cryptococcus neoformans* catalase gene family and its role in antioxidant defense. Eukaryot Cell **5:**1447-1459.
