# Supplementary material for: Unraveling Fungal Radiation Resistance Regulatory Networks through the Genome-Wide Transcriptome and Genetic Analyses of Cryptococcus neoformans
Source: mBio. 2016 Nov 29;7(6):e01483-16. doi: 10.1128/mBio.01483-16 (PMC5137497; doi:10.1128/mBio.01483-16)
Supplement: Table S2 — Primers used in this study. [file mbo006163087st2.pdf]

Table S2. Primers used in this study

Primer Name	Sequence (5'—3')	Comment
B79	TGTGGATGCTGGCGGAGGATA	Screening primer on *ACT1* promter
B1026	GTAAAACGACGGCCAGTGAGC	M13 forward (extended)
B1027	CAGGAAACAGCTATGACCATG	M13 reverse (extended)
B1454	AAGGTGTTCCCCGACGACGAATCG	NSL2
B1455	AACTCCGTCGCGAGCCCCATCAAC	NSR2
B1886	TGGAAGAGATGGATGTGC	NSL-NEO
B1887	ATTGTCTGTTGTGCCCAG	NSR-NEO
B679	CGCCCTTGCTCCTTCTTCTATG	*ACT1* qRT primer 1
B680	GACTCGTCGTATTCGCTCTTCG	*ACT1* qRT primer 2
J61	TTCCCGCCTCACTTCAATC	*ERG1* (CNAG_06829) qRT primer 1
J62	AGGAAGACCCTGGATGGAG	*ERG1* (CNAG_06829) qRT primer 2
B1720	ATCCCTTTTCACCGTCGCTC	*ERG3* (CNAG_00519) qRT primer 1
B6839	GTGATGTTCTAATCTTCTA	*ERG3* (CNAG_00519) qRT primer 2
B671	GTTTTGTTGCCTGAGAACTGGG	*ERG5* (CNAG_06644) qRT primer 1
B672	GTAGATACTGAGAGCCTGCTTGGTG	*ERG5* (CNAG_06644) qRT primer 2
B677	AATCTCCTTACCAGCCATTCGG	*ERG11* (CNAG_00040) qRT primer 1
B678	TTCAGGGAACTTGGGAACAGC	*ERG11* (CNAG_00040) qRT primer 2
J1	TGGTGGTGAGGGAAAATG	*RAD51* (CNAG_00720) qRT primer 1
J2	GCACCTCTTCACCATCAAG	*RAD51* (CNAG_00720) qRT primer 2
J3	AAAGCGATGAGGATGACC	*RDH54* (CNAG_02771) qRT primer 1
J4	CTCGTCTTTTTCAGCACG	*RDH54* (CNAG_02771) qRT primer 2
J5	GCAAAATCTTTCAGCGTGTG	*RAD54* (CNAG_01163) qRT primer 1
J6	CACAAAGTTTCGGGGTTG	*RAD54* (CNAG_01163) qRT primer 2
J11	ACTGAAGCCCAAGTCCACC	*PSO2* (CNAG_03160) qRT primer 1
J12	CCTAAGACTCGGTCGAAATGG	*PSO2* (CNAG_03160) qRT primer 2
J47	AGGCCCTTGAACGAGAAGC	*RDH54* (CNAG_02771) –5' screening primer
J48	AGAGCCGAGACATTGTGAG	*RDH54* (CNAG_02771)– left flanking primer 1
J49	TCACTGGCCGTCGTTTTACTGAAGGTGAAGGATGGCATG	*RDH54* (CNAG_02771) – left flanking primer 2
J50	CATGGTCATAGCTGTTTCCTGGATGCTTGTATGCGGCAC	*RDH54* (CNAG_02771) – right flanking primer 1
J51	TTCCCAAGCAGCAGCTCTC	*RDH54* (CNAG_02771) – right flanking primer 2
J52	TCACTGGTTCCCATCCTCG	*RDH54* (CNAG_02771) – probe primer for Southern blot
J29	CAGCAAGAATGAAATCGGGTC	*RAD54* (CNAG_01163) –5' screening primer
J30	GCCCAAGTGTAAATGAGACG	*RAD54* (CNAG_01163) – left flanking primer 1
J31	TCACTGGCCGTCGTTTTACTGGAGATGATGCCGTAGC	*RAD54* (CNAG_01163) – left flanking primer 2
J32	CATGGTCATAGCTGTTTCCTGTTGAGAGCGGAATTGGGTC	*RAD54* (CNAG_01163) – right flanking primer 1
J33	TCCTAGTTCTGGCAGTGACC	*RAD54* (CNAG_01163) – right flanking primer 2
J34	TGGTCGTCCATAGGATTGTG	*RAD54* (CNAG_01163) – probe primer for Southern blot
J53	TCACCTTCTTCCTCGTCATC	*PSO2* (CNAG_03160) –5' screening primer
J54	AGCTGCTGTTCCTCAGTC	*PSO2* (CNAG_03160) – left flanking primer 1
J55	TCACTGGCCGTCGTTTTACAACTAAGGAGCGTCCCAC	*PSO2* (CNAG_03160) – left flanking primer 2
J56	CATGGTCATAGCTGTTTCCTGCGCGACGAAACATACGTG	*PSO2* (CNAG_03160) – right flanking primer 1
J57	TTCCCTAGCGGCAGTTATC	*PSO2* (CNAG_03160) – right flanking primer 2
J58	GATCTTGTGTCCTCGCACC	*PSO2* (CNAG_03160) – probe primer for Southern blot
C53	CTCTGAGGACGACAAGGACA	*KAR2* (CNAG_06443) qRT primer 1
C54	AGCTCAGAAAGCTGCTCCTC	*KAR2* (CNAG_06443) qRT primer 2
J276	ATGAAGTCGCAGAGGAGC	*LHS1* (CNAG_03899) qRT primer 1
J277	TATCCCTCGCACGGTACTC	*LHS1* (CNAG_03899) qRT primer 2
J278	CGTCTACGGTGATGAGTCC	*PDI1* (CNAG_06240) qRT primer 1
J279	CTCACCAAGAGTGTCCCAG	*PDI1* (CNAG_06240) qRT primer 2
J280	ACACTCTCGCCTTACACATTC	*SCJ1* (CNAG_05252) qRT primer 1
J281	ACATCGCCCGCTTCCATATC	*SCJ1* (CNAG_05252) qRT primer 2
J76	ATTGGGCGCAGGCTTATG	*UBC1* (CNAG_02906) qRT primer 1
J77	ATGTTCCTCAGCCAGTCC	*UBC1* (CNAG_02906) qRT primer 2
J78	CCCAGCTTACACAAACAACAC	*UBC6* (CNAG_05765) qRT primer 2
J79	CCATCGCCAACTGAACAAC	*UBC6* (CNAG_05765) qRT primer 2

J80	TTCTGAAGCTGAGCACTTGG	*UBC62* (CNAG_02214) qRT primer 1
J81	ATCTGTGGCTTCCGCTTG	*UBC62* (CNAG_02214) qRT primer 2
J82	CCACTCCTGCATCCCAATATC	*UBC7* (CNAG_02238) qRT primer 1
J83	CGTTCGGAAGCAGACTCATAC	*UBC7* (CNAG_02238) qRT primer 2
J220	TAACACTCAGCGCCATCC	*RIG1* (CNAG_04055) qRT primer 1
J221	CACATCGGCGTTCATCTCG	*RIG1* (CNAG_04055) qRT primer 2
J198	ATTCGGGTGTAAAGCCTG	*RIG2* (CNAG_03659) qRT primer 1
J199	AGAGGCTGGAATAGACGAGG	*RIG2* (CNAG_03659) qRT primer 2
J218	AGACTGATCGCCAAGGTG	*RIG3* (CNAG_03813) qRT primer 1
J219	ATGTGCATGTCCCGAGGTAG	*RIG3* (CNAG_03813) qRT primer 2
J230	ATACCGATCAGCCCATAGAC	*RIG1* (CNAG_04055) –5' screening primer
J231	TGGAGAGGAAAGAAGTGAGG	*RIG1* (CNAG_04055) – left flanking primer 1
J232	TCACTGGCCGTCGTTTTACAATGTGATGACGGGCGTTG	*RIG1* (CNAG_04055) – left flanking primer 2
J233	CATGGTCATAGCTGTTTCCTGGAACGCCGATGTGACAACC	*RIG1* (CNAG_04055) – right flanking primer 1
J234	GAAGTTGACTCTGGCGAGG	*RIG1* (CNAG_04055) – right flanking primer 2
J235	CATCCGCATACCCGTCATC	*RIG1* (CNAG_04055) – probe primer for Southern blot
J200	CGCCCTATGTTGGATAATC	*RIG2* (CNAG_03659) –5' screening primer
J201	GCACAAGCGGAATAGACTC	*RIG2* (CNAG_03659) – left flanking primer 1
J202	TCACTGGCCGTCGTTTTACACACCGACTTGCCACTTAC	*RIG2* (CNAG_03659) – left flanking primer 2
J203	CATGGTCATAGCTGTTTCCTGAGAAGACTGGCAGAGAATCC	*RIG2* (CNAG_03659) – right flanking primer 1
J204	TTCCACCAATAACGCTCG	*RIG2* (CNAG_03659) – right flanking primer 2
J205	AATCAGGAATCTACGACGG	*RIG2* (CNAG_03659) – probe primer for Southern blot
J236	ACCGGCAGAGTAATCGTC	*RIG3* (CNAG_03813) –5' screening primer
J237	TCCCGTGAGCAACACGATG	*RIG3* (CNAG_03813) – left flanking primer 1
J238	TCACTGGCCGTCGTTTTACACGCTTTACTTGTGCCATG	*RIG3* (CNAG_03813) – left flanking primer 2
J239	CATGGTCATAGCTGTTTCCTGCTTAGGTCAGTGTCGCCTC	*RIG3* (CNAG_03813) – right flanking primer 1
J240	TCAACAATCCGTCCGCAAC	*RIG3* (CNAG_03813) – right flanking primer 2
J241	GTCCTGCAATCACAACTGG	*RIG3* (CNAG_03813) – probe primer for Southern blot
J254	ATATCAACCGCCGATCAGCAC	*BDR1* (CNAG_02589) - qRT primer 1
J255	TCCCTAATTCGTTGTTCACGCAC	*BDR1* (CNAG_02589) - qRT primer 2
J282	GCGGGTAGACATCAAATGC	*BDR1* (CNAG_02589) –5' screening primer
J283	TAGCGCCCAAACCAATCC	*BDR1* (CNAG_02589) – left flanking primer 1
J270	TCACTGGCCGTCGTTTTACAGAAACTCTTCCACACGATG	*BDR1* (CNAG_02589) – left flanking primer 2
J271	CATGGTCATAGCTGTTTCCTGCGTTCTACTGGGAATGATGG	*BDR1* (CNAG_02589) – right flanking primer 1
J272	CATCAAACTCCTCAAACCC	*BDR1* (CNAG_02589) – right flanking primer 2
J273	ATTCGTTGTTCACGCACG	*BDR1* (CNAG_02589) – probe primer for Southern blot
J299	GCGGCCGCACTAACCGCGCAAAATACCT	*BDR1* (CNAG_02589) – primer 1 for complementation
J300	GCGGCCGCCTCATTGATCTCTGTGATGT	*BDR1* (CNAG_02589) – primer 2 for complementation
J301	TGTTCTGCCACTCGCTTG	*BDR1* (CNAG_02589) – primer 1 for sequencing
J302	CGATGTGCTGATTCCCATTC	*BDR1* (CNAG_02589) – primer 2 for sequencing
J303	AGGAGAGATGGACATTGACAAC	*BDR1* (CNAG_02589) – primer 3 for sequencing
J304	TTTGGAGTCAACAGCCGTGG	*BDR1* (CNAG_02589) – primer 4 for sequencing
J319	GGATCCGCGGCCGCCTCATTGATCTCTGTGATGTT	*BDR1* (CNAG_02589) – terminator primer 1 for cloning
J320	AGCTCCTCGCCCTTGCTCACCATTTCCAAGACCGATGATTAC	*BDR1* (CNAG_02589) – promoter primer 1 for cloning
J323	GGATCCCCAACCCCGCTTTCTAGAC	*BDR1* (CNAG_02589) – exon primer 1 for cloning
J321	ATGGTGAGCAAGGGCGAGGAGCT	Primer 1 for GFP cloning
J322	GGATCCAGAGCCACCGCCACCCTTGTACAGCTCGTCCATGC	Primer 2 for GFP cloning
J311	GATGGTCATCGCCGACTTG	*BDR1* (CNAG_02589) –5' screening primer for overexpression
J312	GGCAATGCCGACTCTATCC	*BDR1* (CNAG_02589) – left flanking primer 1 for overexpression
J313	CACTCGAATCCTGCATGCTTCCAAGACCGATGATTACA	*BDR1* (CNAG_02589) – left flanking primer 2 for overexpression
J314	ACCACAACACATCTATCACATGCCAACCCCGCTTTCTAG	*BDR1* (CNAG_02589) – right flanking primer 1 for overexpression
J273	ATTCGTTGTTCACGCACG	*BDR1* (CNAG_02589) – right flanking primer 2 for overexpression
J603	ATATCAACCGCCGATCAGC	*BDR1* (CNK00770) - qRT primer 1 for serotype D JEC21
J604	TTCCCTGATTCGCTGTTCAC	*BDR1* (CNK00770) - qRT primer 2 for serotype D JEC21
J605	AACGGATTTCTCGGGTCG	*RAD51* (CNA06990) - qRT primer 1 for serotype D JEC21
J606	CAGCAACGCCAAACTCATC	*RAD51* (CNA06990) - qRT primer 2 for serotype D JEC21
J607	GGCAGCAGAATTGAGGGAC	*RDH54* (CNC03920) - qRT primer 1 for serotype D JEC21
J608	TCCCTCGTTTCGCCTACAG	*RDH54* (CNC03920) - qRT primer 2 for serotype D JEC21

J609	GTCGCAGTAAGCGGTATGG	*RAD54* (CND02890) - qRT primer 1 for serotype D JEC21
J610	TTCCTTGCTTTCGGGCTG	*RAD54* (CND02890) - qRT primer 2 for serotype D JEC21
J611	TAACACTCAGCGCCATC	*RIG1* (CNB05390) - qRT primer 1 for serotype D JEC21
J612	GGTTGTTACATCGGCGTTC	*RIG1* (CNB05390) - qRT primer 2 for serotype D JEC21
J613	TGATGTGTTAAGGCGGCTG	*RIG2* (CNB01590) - qRT primer 1 for serotype D JEC21
J614	AGCGGGAGTTTGTGCTCTTG	*RIG2* (CNB01590) - qRT primer 2 for serotype D JEC21
J615	CAGCAACCCTTGAGACAAGC	*RIG3* (CNB03040) - qRT primer 1 for serotype D JEC21
J616	TTTGACTGTCCCGATGATCTC	*RIG3* (CNB03040) - qRT primer 2 for serotype D JEC21
J170	CGCAGGTCTCGAATCTGTC	*ATG4* (CNAG_02662) - qRT primer 1
J171	TCGTTGACGGGAGAGGAAG	*ATG4* (CNAG_02662) - qRT primer 2
J172	CACAACTTGCGAGCGTAC	*ATG3* (CNAG_06892) - qRT primer 1
J173	GGTTCAGTAATAGGCGCTGG	*ATG3* (CNAG_06892) - qRT primer 2
J176	AGGATTCCCGTGATCTGTG	*ATG8* (CNAG_00816) - qRT primer 1
J177	GACGAATTGCCCAACAGTAAG	*ATG8* (CNAG_00816) - qRT primer 2
J182	ATTCCAGAGGGGTGAGAGAG	*ATG8* (CNAG_00816) –5' screening primer
J183	ATCTGAGACTGATGGTGGG	*ATG8* (CNAG_00816) – left flanking primer 1
J184	TCACTGGCCGTCGTTTTACATGGGATGTGAGATAGCGG	*ATG8* (CNAG_00816) – left flanking primer 2
J185	CATGGTCATAGCTGTTTCCTGCAGCGGCACTTATGAGTTC	*ATG8* (CNAG_00816) – right flanking primer 1
J186	ACCGTGATTGTGTTCGTG	*ATG8* (CNAG_00816) – right flanking primer 2
J187	TCTTCTCAGCCTTCTCACAG	*ATG8* (CNAG_00816) – probe primer for Southern blot
J426	TTCCGGCTCCATCTCATC	*ATG3* (CNAG_06892) –5' screening primer
J427	ATGCGGAGAGGATGATCG	*ATG3* (CNAG_06892) – left flanking primer 1
J428	TCACTGGCCGTCGTTTTACAAGTCTCTTGCCCTGGATG	*ATG3* (CNAG_06892) – left flanking primer 2
J429	CATGGTCATAGCTGTTTCCTGTCGACCGTATGGAAGCTG	*ATG3* (CNAG_06892) – right flanking primer 1
J430	CATCAACTTCCAGCACAACC	*ATG3* (CNAG_06892) – right flanking primer 2
J441	TGGGATGCACAATCCTCAC	*ATG3* (CNAG_06892) – probe primer for Southern blot
J431	CCCTCAATGGATGCTGGAC	*ATG4* (CNAG_02662) –5' screening primer
J432	CCATCTGGAAGAGGAAGACC	*ATG4* (CNAG_02662) – left flanking primer 1
J433	TCACTGGCCGTCGTTTTACAACGCTGAGGTAATGCTGC	*ATG4* (CNAG_02662) – left flanking primer 2
J434	CATGGTCATAGCTGTTTCCTGATTGGAAGAGCCCGGAAC	*ATG4* (CNAG_02662) – right flanking primer 1
J435	GAAGATGCACGCACGGAAG	*ATG4* (CNAG_02662) – right flanking primer 2
J442	AAGACTGGTAGGCGATGG	*ATG4* (CNAG_02662) – probe primer for Southern blot
B865	ATTCATCGGGGGGTTATC	*CAT1* (CNAG_04981) –5' screening primer
B861	AACGGGAATGGGGAAGTCTC	*CAT1* (CNAG_04981) – left flanking primer 1
B862	CTGGCCGTCGTTTTACCCTTGGTTCAGCATTTGC	*CAT1* (CNAG_04981) – left flanking primer 2
B863	GTCATAGCTGTTTCCTGTCCGTTCAGTTTGCTAAGC	*CAT1* (CNAG_04981) – right flanking primer 1
B864	ATGTTGCCTTTGGGTGTC	*CAT1* (CNAG_04981) – right flanking primer 2
B1925	GCAAGCAACCCTTTTCTG	*CAT1* (CNAG_04981) – probe primer for Southern blot
B859	CATTTGACATTACAGGGTGG	*CAT2* (CNAG_05256) –5' screening primer
B855	CGTCCCACAAACATTCTTAC	*CAT2* (CNAG_05256) – left flanking primer 1
B856	CTGGCCGTCGTTTTACGTATGTGGGTGTTTTCTCAGTC	*CAT2* (CNAG_05256) – left flanking primer 2
B857	GTCATAGCTGTTTCCTGGCTCAACAAGGGAAAGTAAAGG	*CAT2* (CNAG_05256) – right flanking primer 1
B858	TGATACCGCAACCAGTTCC	*CAT2* (CNAG_05256) – right flanking primer 2
B860	AAGAGTCGGCGGTGTGTTTC	*CAT2* (CNAG_05256) – probe primer for Southern blot
B871	ATGCGGAAAAGCCTGTAG	*CAT3* (CNAG_00575) –5' screening primer
B867	ACAAGGGGTCGTTCAATAG	*CAT3* (CNAG_00575) – left flanking primer 1
B868	CTGGCCGTCGTTTTACACCTTGACCCCGAAATACG	*CAT3* (CNAG_00575) – left flanking primer 2
B869	GTCATAGCTGTTTCCTGCATAGGGTTTGGGACAGAG	*CAT3* (CNAG_00575) – right flanking primer 1
B870	TGGACGAGAAGAAGAGCAC	*CAT3* (CNAG_00575) – right flanking primer 2
B872	ATCCCAGTTTCCTTCGTCCG	*CAT3* (CNAG_00575) – probe primer for Southern blot
B853	TTCAAGCCCGTAGCACACAGGTTC	*CAT4* (CNAG_05015) –5' screening primer
B849	CGTTGGCAAGGAAGTGATG	*CAT4* (CNAG_05015) – left flanking primer 1
B850	CTGGCCGTCGTTTTACTACCAGAGGCAAGGTTACG	*CAT4* (CNAG_05015) – left flanking primer 2
B851	GTCATAGCTGTTTCCTGTGGTGCTCCTCAGAAGACTC	*CAT4* (CNAG_05015) – right flanking primer 1
B852	TGATTTGACCAACACTCCG	*CAT4* (CNAG_05015) – right flanking primer 2
B854	GGTCCCTATCCTTCTCGTATG	*CAT4* (CNAG_05015) – probe primer for Southern blot
J389	GCCACTCAAGACTGATCCTAC	*SRX1* (CNAG_00654) - qRT primer 1
J390	CGTTTCCCATCCCAGTCTC	*SRX1* (CNAG_00654) - qRT primer 2
J391	GATGTTGAGGAGCAAGAGGAC	*TRX1* (CNAG_02801) - qRT primer 1
J392	CCGGTAACAGTCTCGATCAC	*TRX1* (CNAG_02801) - qRT primer 2

J393	GCCGTGTTCTCACTCCTTTAC	*TRX2* (CNAG_01607) - qRT primer 1
J394	CTTGGCAGCAACTTCAGG	*TRX2* (CNAG_01607) - qRT primer 2
J395	CATGGACTTCACCTTCGTTTG	*TSA1* (CNAG_03482) - qRT primer 1
J396	CGGTGGAGACACAGATGAC	*TSA1* (CNAG_03482) - qRT primer 2
J397	AAGCAGATCCGTCTCACC	*TSA3* (CNAG_06917) - qRT primer 1
J398	CGGTGTTTATCGCCAAGC	*TSA3* (CNAG_06917) - qRT primer 2
J399	ACCAACGGCTGTACCTCTG	*SOD1* (CNAG_01019) - qRT primer 1
J400	ATATTACCGAGGTCACCAACG	*SOD1* (CNAG_01019) - qRT primer 2
J401	CAAGGCCACCAAGAAGCTC	*SOD2* (CNAG_04388) - qRT primer 1
J402	TAGAAAGCGTGCTCCCAG	*SOD2* (CNAG_04388) - qRT primer 2
J403	TTATTCACGCGGTAAGGGAG	*CAT1* (CNAG_04981) - qRT primer 1
J404	GAAATCTCCAGGCAAGCAAG	*CAT1* (CNAG_04981) - qRT primer 2
J405	ACCGAGACAACGGATGAGG	*CAT2* (CNAG_05256) - qRT primer 1
J406	TGACACGAGACATTGGTGATC	*CAT2* (CNAG_05256) - qRT primer 2
J407	TGCCTTCGTATCCAAGCTC	*CAT3* (CNAG_00575) - qRT primer 1
J408	ATTGCGTAGCATTGCCGAC	*CAT3* (CNAG_00575) - qRT primer 2
J409	ATCGCCTGATCTTGGAGC	*CAT4* (CNAG_05015) - qRT primer 1
J410	CGCCAAAGCCATTATGAGTAC	*CAT4* (CNAG_05015) - qRT primer 2
J96	TCTGGATACAGGGCGGAAG	*LAC1* (CNAG_03465) - qRT primer 1
J97	TCCCTCAGTAAGATGCCAC	*LAC1* (CNAG_03465) - qRT primer 2
J98	GTTTCAGTTGATGGGACGAGG	*LAC2* (CNAG_03464) - qRT primer 1
J99	TCCAAATCGTATCCTTCCTCG	*LAC2* (CNAG_03464) - qRT primer 2
